# A single pair of leucokinin neurons are modulated by feeding state and regulate sleep–metabolism interactions

**DOI:** 10.1371/journal.pbio.2006409

**Published:** 2019-02-13

**Authors:** Maria E. Yurgel, Priyanka Kakad, Meet Zandawala, Dick R. Nässel, Tanja A. Godenschwege, Alex C. Keene

**Affiliations:** 1 Department of Biological Sciences, Florida Atlantic University, Jupiter, Florida, United States of America; 2 Integrative Biology and Neuroscience Graduate Program, Florida Atlantic University, Jupiter, Florida, United States of America; 3 Department of Zoology, Stockholm University, Stockholm, Sweden; Washington University School of Medicine, United States of America

## Abstract

Dysregulation of sleep and feeding has widespread health consequences. Despite extensive epidemiological evidence for interactions between sleep and metabolic function, little is known about the neural or molecular basis underlying the integration of these processes. *D*. *melanogaster* potently suppress sleep in response to starvation, and powerful genetic tools allow for mechanistic investigation of sleep–metabolism interactions. We have previously identified neurons expressing the neuropeptide leucokinin (Lk) as being required for starvation-mediated changes in sleep. Here, we demonstrate an essential role for Lk neuropeptide in metabolic regulation of sleep. The activity of Lk neurons is modulated by feeding, with reduced activity in response to glucose and increased activity under starvation conditions. Both genetic silencing and laser-mediated microablation localize Lk-dependent sleep regulation to a single pair of Lk neurons within the Lateral Horn (LHLK neurons). A targeted screen identified a role for 5′ adenosine monophosphate-activated protein kinase (AMPK) in starvation-modulated changes in sleep. Knockdown of AMPK in Lk neurons suppresses sleep and increases LHLK neuron activity in fed flies, phenocopying the starvation state. Further, we find a requirement for the Lk receptor in the insulin-producing cells (IPCs), suggesting LHLK–IPC connectivity is critical for sleep regulation under starved conditions. Taken together, these findings localize feeding-state–dependent regulation of sleep to a single pair of neurons within the fruit fly brain and provide a system for investigating the cellular basis of sleep–metabolism interactions.

## Author summary

Neural regulation of sleep and feeding are interconnected and are critical for survival. Many animals reduce their sleep in response to starvation, presumably to forage for food. Here, we find that in the fruit fly *Drosophila melanogaster*, the neuropeptide leucokinin is required for the modulation of starvation-dependent changes in sleep. Leucokinin is expressed in numerous populations of neurons within the two compartments of the central nervous system: the brain and the ventral nerve cord. Both genetic manipulation and laser-mediated microablation experiments identify a single pair of neurons expressing this neuropeptide in the brain as being required for metabolic regulation of sleep. These neurons become active during periods of starvation and modulate the function of insulin-producing cells that are critical modulators of both sleep and feeding. Supporting this notion, knockdown of the leucokinin receptor within the insulin-producing cells also disrupts metabolic regulation of sleep. Taken together, these findings identify a critical role for leucokinin signaling in the integration of sleep and feeding states.

## Introduction

Dysregulation of sleep and feeding has widespread health consequences, and reciprocal interactions between these processes underlie a number of pathologies [1–4]. Sleep loss correlates with increased appetite and insulin insensitivity, while short-sleeping individuals are more likely to develop obesity, metabolic syndrome, type 2 diabetes, and cardiovascular disease [1,3,4]. Although the neural basis for sleep regulation has been studied in detail, little is known about how feeding state and changes in metabolic function modulate sleep [5,6]. Understanding how sleep and feeding states are integrated may provide novel insights into the comorbidity of disorders linked to sleep and metabolic regulation.

Animals balance nutritional state and energy expenditure in order to achieve metabolic homeostasis [6,7]. In both flies and mammals, diet potently affects sleep regulation, supporting the notion that sleep and metabolic state interact [5,6,8]. Starvation leads to sleep loss or disrupted sleep architecture, presumably to induce foraging behavior, while high-calorie diets have complex effects on sleep depending on macronutrient content [9–12]. Behavioral and physiological responses to changes in feeding state are modulated both by cell-autonomous nutrient centers in the brain that detect changes in circulating nutrients and through communication between brain and peripheral tissues [13], yet the neural basis for the integration of sleep and feeding state remain poorly understood.

The fruit fly, *D*. *melanogaster*, provides a powerful model for investigating sleep regulation. Flies display all the behavioral hallmarks of sleep, including extended periods of behavioral quiescence, rebound following deprivation, increased arousal threshold, and species-specific changes in posture [14,15]. Many genetic mechanisms regulating sleep are conserved from flies to mammals. In addition, high-throughput systems for sleep analysis in *Drosophila* have led to the identification of both novel and highly conserved sleep genes [16,17]. Further, stimulants including caffeine, amphetamine, and cocaine have been shown to suppress sleep in flies [15,18,19]. Thus, at the molecular, pharmacological, and behavioral levels, flies provide a model for studying genetic regulation of mammalian sleep.

A number of genes and neurons that are required for the integration of sleep and feeding states have been identified, including core-circadian clock genes, metabolic hormones, and sensory neurons [9,20–22]. While many identified regulators of sleep–metabolism interactions broadly impact these processes [6], a mutation of the DNA/RNA binding protein *translin* (*trsn*) disrupts starvation-induced sleep suppression without affecting sleep or metabolic regulation under fed conditions. Targeted knockdown in approximately 30 leucokinin (Lk) neurons phenocopies *trsn* mutants, raising the possibility that these neurons are required for the integration of sleep and metabolic state [23].

Here, we identify a single pair of Lk neurons in the lateral horn of the fly brain that are required for the integration of sleep and metabolic state. These neurons project near the insulin-producing cells (IPCs), which are critical modulators of sleep and metabolic regulation [24–26]. Lateral Horn leucokinin (LHLK) neurons are dispensable for sleep under fed conditions but are required for starvation-induced sleep suppression. Functional imaging reveals that LHLK neurons have reduced activity in response to glucose application and increased activity under starved conditions. The identification of single neurons that integrate sleep and metabolic state provide a model for investigating the cellular mechanisms regulating the integration of sleep and metabolic state.

## Results

**Fig 1:**
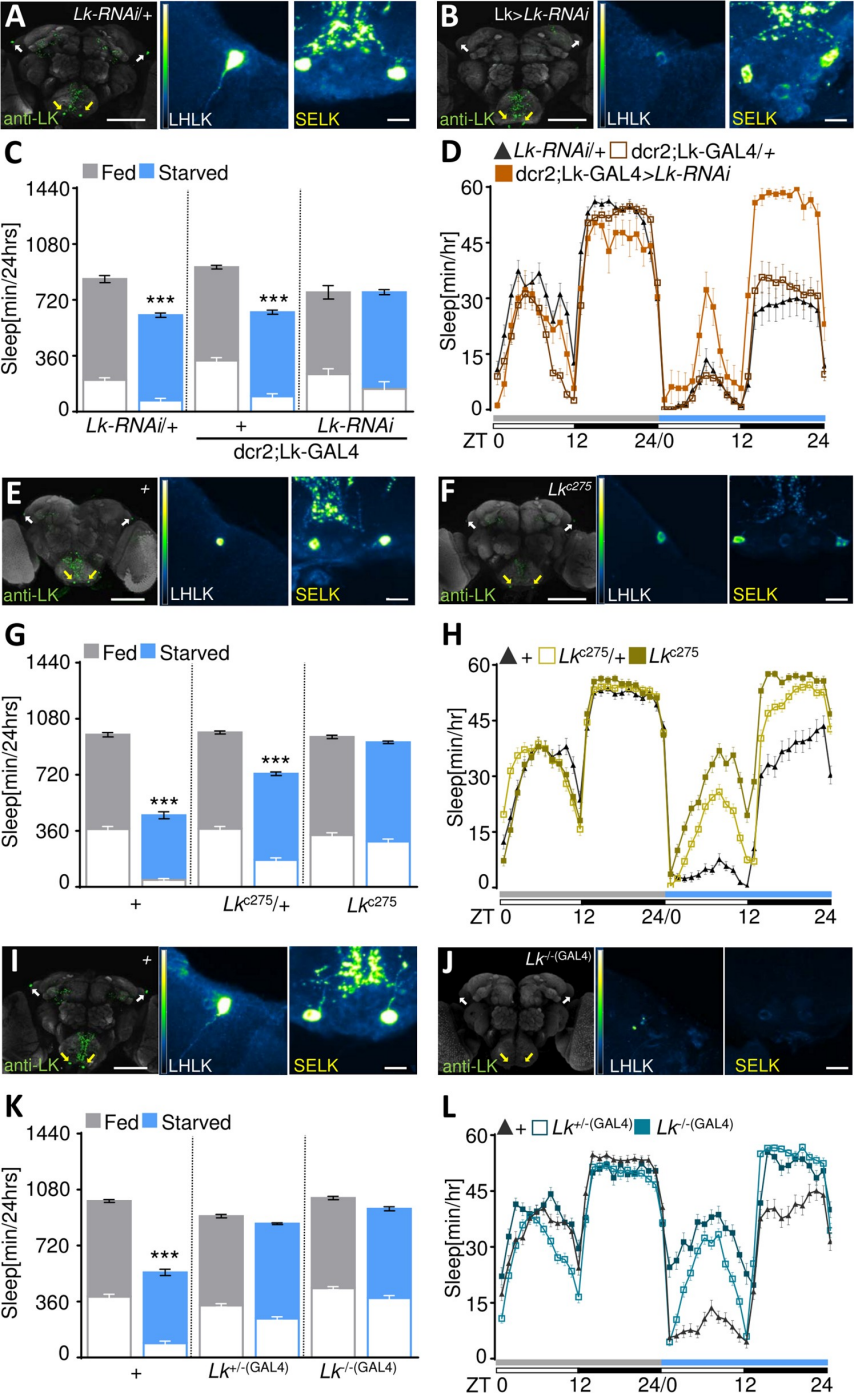
Lk is required for metabolic regulation of sleep. (A, B) IHC using antibody against Lk. White arrows indicate LHLK cell bodies, and yellow arrows indicate SELK cell bodies. Lk staining in flies expressing *Lk-RNAi* results in loss of Lk protein level in LHLK neurons (B) compared to *UAS-Lk-RNAi*/+ control (A). The brains were counterstained with nc82 (bruchpilot, gray). Scale bar = 100 μm. Color-coded images reflect fluorescence intensity, where minimum = 0 and max = 4,025. Scale bar for color-coded images = 10 μm. (C) Sleep is significantly reduced in starved *UAS-Lk-RNAi*/+ (*n* ≥ 20, *p* < 0.0001) and dcr2,Lk-GAL4/+ (*n* ≥ 25, *p* < 0.0001) control flies, while no significant differences are observed in Lk-GAL4>UAS-dcr2,*Lk-RNAi* flies (*n* = 13, *p* > 0.99). Two-way ANOVA (F [2, 141] = 10.87). White bars in column graphs represent amount of sleep during the day (ZT 0–12), while colored bars represent night sleep (ZT 12–24). (D) Sleep profile representative of (C). Flies are placed in food tubes during day 1 (fed, gray), then transferred to agar during day 2 (starved, blue). White/black bars represent lights on and off, respectively. (E, F) Lk staining in *Lk*^*c275*^ mutants reveals reduced protein levels compared to *w*^*1118*^ control (E). (G) Sleep is significantly reduced in starved *w*^*1118*^ controls (*n* = 63, *p* < 0.0001) and flies harboring one copy of *Lk*^*c275*^ (*n* = 68, *p* < 0.0001), while no significant differences are observed in *Lk*^*c275*^ mutants (*n* = 58, *p* = 0.90). Two-way ANOVA (F [2, 372] = 53.57). (H) Sleep profile representative of data in (G). (I, J) Lk protein levels are reduced in *Lk*^*−/−(GAL4)*^ flies compared to *w*^*1118*^ control (J). (K) *Lk*^*−/− (GAL4)*^ (*n* ≥ 47; *p* = 0.31) mutant flies and *Lk*^*+/−(GAL4)*^ (*n* = 68, *p* = 0.35) fail to suppress sleep in response to starvation, while *w*^*1118*^ control flies suppress sleep (*n* = 75, *p* < 0.0001). Two-way ANOVA (F [2, 376] = 57.03). (L) Sleep profile representative of data in (K). All columns represent mean ± SEM; *p < 0.05; **p < 0.01; ***p < 0.001. Underlying data can be found in S1 Data. ANOVA, analysis of variance; dcr2, dicer-2; GAL4, galactose-responsive transcription factor; IHC, immunohistochemistry; LHLK, Lateral Horn leucokinin; Lk, leucokinin; max, maximum; nc82, neuropil marker; RNAi, RNA interference; SELK, subesophageal ganglion leucokinin; UAS, upstream activation sequence; ZT, Zeitgeber time.

Leucokinin (Lk) neuropeptide has been implicated in regulation of feeding, sleep, and circadian activity [27,28]. To determine whether Lk is required for metabolic regulation of sleep, we measured sleep under fed and starved conditions in mated female flies with disrupted Lk expression. In agreement with previous findings, driving *Lk-RNAi* (RNA interference) under control of Lk-galactose-responsive transcription factor (GAL4) (yeast transcription factor, Lk-GAL4>upstream activation sequence [UAS]-dicer-2 [dcr2],*Lk-RNAi*) significantly reduced Lk expression (Fig 1A and 1B and S1B Fig). Control flies harboring *Lk-RNAi* or Lk-GAL4 transgenes alone significantly suppressed sleep during starvation, while no significant differences were detected between the fed and starved states in Lk>*Lk-RNAi* knockdown flies (Fig 1C and 1D). To confirm that these phenotypes are not due to off-target effects, we measured starvation-induced sleep suppression in two *Lk* mutants. *Lk*^*c275*^ is a hypomorphic allele containing a piggyBac element upstream of the *Lk* gene transcription start site (S1A Fig), with approximately 30% reduction in Lk levels (Fig 1E and 1F and S1B Fig) [28]. Flies homozygous for *Lk*^*c275*^ failed to suppress sleep when starved (Fig 1G and 1H). We also used Clustered Regularly Interspaced Short Palindromic Repeats (CRISPR/Cas9) gene-editing to generate a recombinant transgenic line (*Lk*^−*/*−*(GAL4)*^) by replacing bases 1 to 7 downstream of the *Lk* ATG start codon with a GAL4-element containing cassette (S1A Fig). Lk protein was not detected in the brains of *Lk*^*−/−(GAL4)*^ mutants, confirming that this genetic modification resulted in a robust reduction of Lk function (Fig 1I and 1J and S1B Fig). Sleep while fed did not differ between *Lk*^*−/−(GAL4)*^ lines and *w*^*1118*^ controls, suggesting that *Lk* is not required for sleep regulation under fed conditions (Fig 1K and 1L). Conversely, control *w*^*1118*^ flies, but not *Lk*^*−/−(GAL4)*^ flies, robustly suppressed sleep under starved conditions during the day and night, indicating that *Lk* is required for starvation-induced sleep suppression (Fig 1K and 1L). In agreement with previous findings, starvation induces hyperactivity in control flies (S1C and S1D Fig) [20,29]. Starvation did not alter waking activity in *Lk*^*c275*^ and *Lk*^*−/−(GAL4)*^ flies, indicating that Lk is required for both starvation-induced changes in sleep regulation and hyperactivity (S1C and S1D Fig).

To determine whether the sleep phenotype was caused by loss of *Lk*, we restored *Lk* in the background of each mutant and measured sleep. Pan-neuronal rescue (*Lk*^*c275*^; embryonic lethal abnormal vision [elav]-GAL4/*UAS-Lk*) restored starvation-induced sleep suppression (S1E Fig) and starvation-induced hyperactivity (S1F Fig). Rescue flies did not differ from heterozygous controls (*Lk*^*c275*^; elav-GAL4/+ or *UAS-Lk*;*Lk*^*c275*^/+). The GAL4 insertion in *Lk*^*−/−(GAL4)*^ drives expression to a pattern similar to Lk antibody, providing the opportunity to restore Lk to its endogenous expression pattern (S1G Fig). Similar to *Lk*^*c275*^, rescue in Lk neurons (*Lk*^*−/−(GAL4)*^; *UAS-Lk)* restored starvation-induced sleep suppression (S1H Fig) and starvation-induced hyperactivity (S1I Fig), confirming that *Lk* is required for the metabolic regulation of sleep. Flies heterozygous for *Lk*^*−/−(GAL4)*^ failed to suppress sleep in response to starvation (Fig 1K) and displayed reduced starvation-induced hyperactivity compared to control (S1D Fig), raising the possibility that haploinsufficiency impacts sleep regulation. This result is consistent with the notion that the moderate reduction in Lk levels in *Lk*^*c275*^ mutants can affect diverse behavioral phenotypes [27]. Expression of a rescue transgene in Lk neurons of flies heterozygous for *Lk* (*Lk*^*+/−(GAL4)*^;*UAS-Lk*) also restored starvation-induced sleep suppression (S1J Fig), confirming the specificity of phenotype in *Lk*^*−/−(GAL4)*^ flies. Taken together, three independent genetic manipulations that perturb *Lk* expression inhibit starvation-induced changes in sleep and activity.

**Fig 2:**
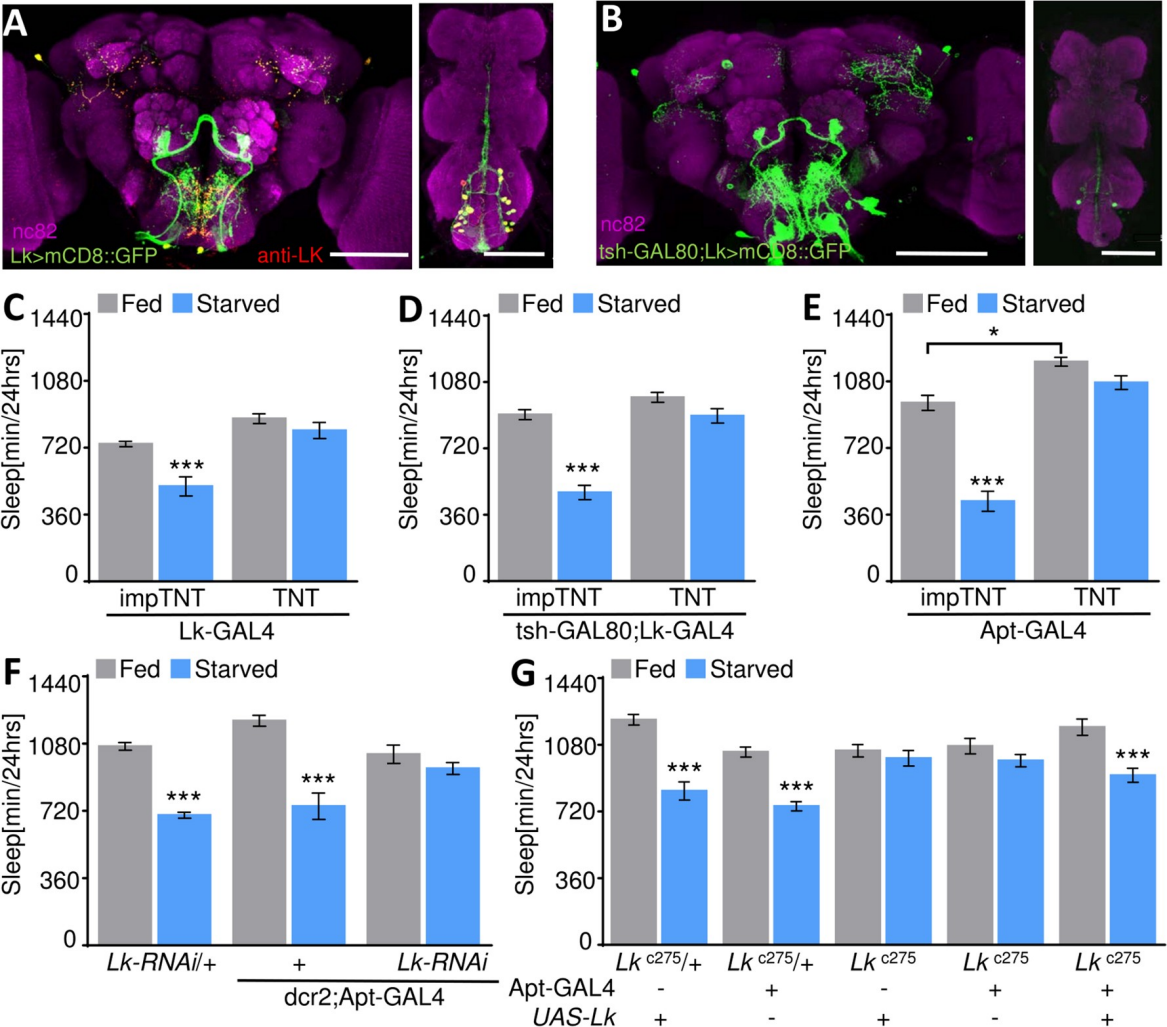
LHLK neurons are required for the metabolic regulation of sleep. (A) Whole-brain and ventral nerve cord confocal reconstruction of Lk-GAL4>CD8::GFP. GFP-expressing neurons (green). Immunostaining for anti-Lk (red) reveals colocalization of LHLK and SELK neurons (yellow). The brain and ventral nerve cord are counterstained with nc82 (magenta). Scale bar = 100 μm. (B) GFP expression in the ventral nerve cord (ABLK neurons) of flies carrying Lk-GAL4>CD8::GFP is blocked by the tsh-GAL80 transgene. (C) Blocking synaptic release in Lk neurons with TNT impairs starvation-induced sleep suppression (*n* = 29, *p* = 0.60), while impTNT controls suppress sleep (*n* ≥ 27, *p* = 0.0002). No differences were observed between genotypes during the fed state (*p* = 0.06). Two-way ANOVA (F [1, 109] = 4.88). (D) Starvation-induced sleep suppression is absent in tsh-GAL80;Lk-GAL4 flies (*n* = 41, *p* = 0.12), while controls expressing impTNT suppress sleep (*n* = 33, *p* < 0.001). Sleep duration while fed does not differ significantly between tsh-GAL80;Lk-GAL4>TNT and impTNT flies (*p* = 0.21). Two-way ANOVA (F [1, 144] = 22.53). (E) TNT expression in Apt-GAL4 neurons abolishes starvation-induced sleep suppression (*n* ≥ 18, *p* = 0.48) and shows a significant increase in sleep during the fed state (*p* = 0.01), compared to impTNT that suppresses sleep (*n* ≥ 34, *p* < 0.0001). Two-way ANOVA (F [1, 103] = 18.22). (F) Expression of *Lk-RNAi* in Apt-GAL4 neurons (Apt-GAL4>UAS-dcr2,*Lk-RNAi*, *n* ≥ 12) blocks starvation-induced sleep suppression (*p* = 0.877), while *UAS-Lk-RNAi*/+ (*n* = 79, *p* < 0.0001) and dcr2,Apt-GAL4/+ (*n* = 13, *p* < 0.0001) controls suppress sleep. Two-way ANOVA (F [2, 201] = 12.44). (G) Expression of *UAS-Lk* under control of Apt-GAL4 in the *Lk*^*c275*^ mutant background restores starvation-induced sleep suppression (*n* = 15, *p* = 0.002) compared to *Lk*^*c275*^ flies and mutant control flies *UAS-Lk*/+;*Lk*^*c275*^ (*n* = 16; *p* = 0.98) or Apt-GAL4/+;*Lk*^*c275*^ (*n* = 16, *p* = 0.88). Control flies, Apt-GAL4/+;*Lk*^*c275*^/+ (*n* ≥ 16, *p* < 0.0001) or *UAS-Lk*/+;*Lk*^*c275*^/+ (*n* = 21, *p* < 0.0001), suppress sleep in response to starvation. Two-way ANOVA (F [4, 159] = 8.13). All columns are mean ± SEM; *p < 0.05; **p < 0.01; ***p < 0.001. Underlying data can be found in S1 Data. ANOVA, analysis of variance; Apt, *Apterous*; CD8::GFP; membrane-tethered GFP, LK-GAL4>CD8:GFP;tshGAL80; dcr2, dicer-2; GAL4, galactose-responsive transcription factor; GFP, green fluorescent protein; impTNT, inactive variant of tetanus toxin; LHLK, Lateral Horn leucokinin; Lk, leucokinin; nc82, neuropil marker; RNAi, RNA interference; TNT, tetanus toxin; tsh, teashirt; UAS, upstream activation sequence.

Leucokinin antibody labels the LHLK neurons and the subesophoageal ganglion Lk (SELK) neurons, as well as a number of abdominal Lk (ABLK) neurons in the ventral nerve cord (Fig 2A). To localize the population of neurons that regulate starvation-induced sleep suppression, we restricted GAL4 expression primarily to the brain through the expression of GAL80, a GAL4 repressor, in the ventral nerve cord using *teashirt*-GAL80 (tsh-GAL80) [30]. Expression of CD8::GFP (Lk-GAL4>CD8:green fluorescent protein [GFP];tsh-GAL80) revealed tsh-GAL80 blocks expression in all but two ventral nerve cord neurons without affecting expression in the brain (Fig 2B). Silencing the remaining Lk neurons with light-chain tetanus toxin (TNT) (tsh-GAL80;Lk-GAL4>UAS-TNT) abolished starvation-induced sleep suppression, phenocopying the effects of silencing all Lk neurons (Lk-GAL4>UAS-TNT) (Fig 2C and 2D) [31]. Further, no differences in sleep were detected between groups in fed flies, and there was no effect of expressing an inactive variant of TNT light chain (impTNT) in Lk neurons (Fig 2C and 2D). These findings suggest Lk neurons within the brain are required for sleep–metabolism interaction.

It has previously been reported that *Apterous*-GAL4 (Apt-GAL4) drives expression in the LHLK neurons, as well as neurons in the optic lobe and antennal mechanosensory and motor centers (AMMCs), and a small population of mushroom-body neurons (S2A Fig) [27,32]. Immunostaining with Lk antibody in Apt-GAL4>UAS-mCD8::GFP flies confirmed colocalization exclusively within the LHLK neurons (S2A and S2B Fig). To functionally assess the role of LHLK neurons, we genetically silenced LHLK neurons, as well as other non-Lk cells labeled by Apt-GAL4. Silencing neurons labeled by Apt-GAL4 (Apt-GAL4>UAS-TNT) inhibited starvation-induced sleep suppression and promoted sleep while fed, while no effects were observed in flies expressing impTNT (Apt-GAL4>UAS-impTNT) (Fig 2E). While these findings suggest a role for Apt-GAL4–labeled neurons in sleep regulation, it is possible that the phenotype is independent of Lk function.

To verify that the sleep phenotype was due to blocking Lk release from LHLK neurons, we sought to disrupt Lk function selectively in Apt-GAL4–labeled neurons. Expression of *Lk-RNAi* in Apt-Gal4 neurons (Apt-GAL4>UAS-dcr2, *Lk-RNAi*) disrupted starvation-induced sleep suppression (Fig 2F). As a complementary approach, we restored Lk to Apt-GAL4 neurons in the *Lk*^*c275*^ mutant background and measured sleep. Rescue in Apt-GAL4 neurons (*Lk*^*c275*^; Apt-GAL4>*Lk*^*c275*^; *UAS-Lk*) restored starvation-induced sleep suppression to heterozygote control levels (Apt-GAL4; *Lk*^*c275*^/+ and *UAS-Lk*; Lk^c275^/+), whereas flies harboring either GAL4 or UAS in the *Lk*^*c275*^ mutant background failed to suppress sleep (Apt-GAL4; Lk^c275^ and *UAS-Lk*; Lk^c275^; Fig 2G). These data support a role for the LHLK neurons in starvation-induced sleep suppression but do not rule out the possibility that other neurons labeled by Apt-GAL4 also contribute to this phenotype.

**Fig 3:**
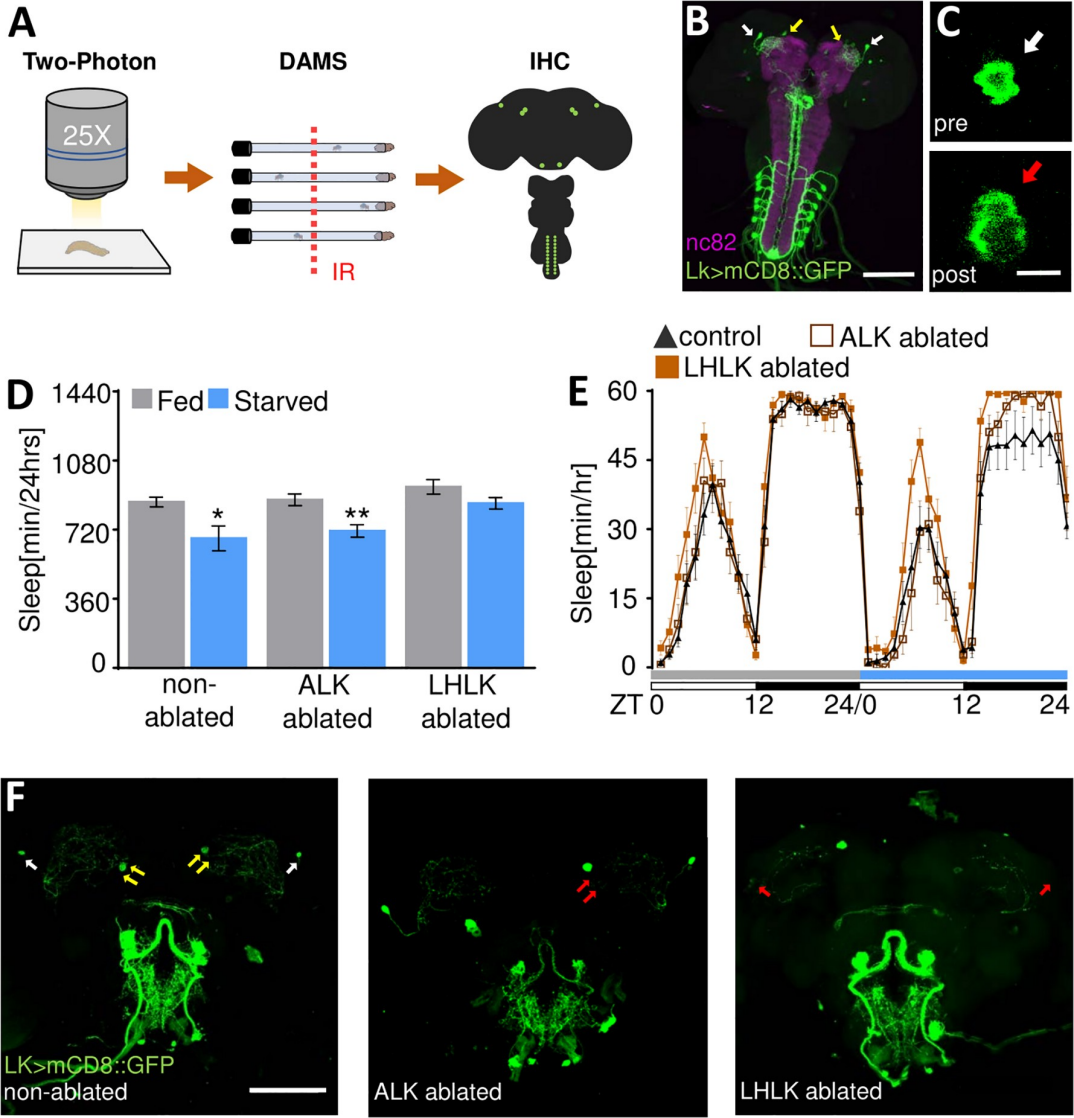
Laser-induced microablation of LHLK neurons. (A) Diagram representative of targeted multiphoton ablation. Third-instar larvae expressing UAS-mCD8::GFP in Lk neurons are placed dorsally onto a microscope slide, and neurons are ablated. Following ablation, larvae are placed in vials containing food and allowed to grow. Sleep in food vials and on agar is measured 5–7 days posteclosion in the DAMS. IHC is performed to verify ablated neurons. (B) Expression pattern of Lk-GAL4 during third-instar larval stage visualized with mCD8::GFP (green). The CNS was counterstained with nc82 (magenta). Scale bar = 100 μm. (C) Representative images of neuron pre- (top, white arrow) and postablation (bottom, red arrow). Scale bar = 10 μm. (D) Flies with bilateral laser ablation of LHLK neurons fail to suppress sleep when starved (*n* = 12, *p* = 0.09, *t* = 1.18), while ablation of a pair of ALK neurons (*n* = 9, *p* = 0.007, *t* = 3.04) and nonablated controls (*n* = 14, *p* = 0.01, *t* = 2.73) suppresses sleep in response to starvation. Unpaired *t* test. (E) Sleep profile representative of (D). Flies are placed in food tubes during day 1 (fed, gray), then transferred to agar during day 2 (starved, blue). White/black bars represent lights on and off, respectively. (F) Representative images of GFP-expressing Lk neurons post-LHLK (right) and ALK ablation (middle) and treated but nonablated controls (left). Red arrows indicate ablated neurons. White and yellow arrows indicate intact LHLK neurons and ALK neurons, respectively. Scale bar = 100 μm. All columns are mean ± SEM; *p < 0.05; **p < 0.01; ***p <0.001. Underlying data can be found in S1 Data. ALK, anterior leucokinin; CNS, central nervous system; DAMS, *Drosophila* Activity Monitor System; GAL4, galactose-responsive transcription factor; GFP, green fluorescent protein; IHC, immunohistochemistry; IR, infrared light; LHLK, Lateral Horn leucokinin; Lk, leucokinin; mCD8, membrane-tethered; nc82, neuropil marker; UAS, upstream activation sequence; ZT, Zeitgeber time.

To complement genetic silencing experiments, we sought to precisely ablate the LHLK neurons and measure their role in starvation-induced sleep suppression (Fig 3A). Multiphoton microscopy has been used in diverse genetic models for targeted ablation of neuronal cell types [33–35]. All adult Lk neurons are present in third-instar larvae and labeled by the Lk-GAL4. The SELK and anterior Lk (ALK) neurons are easily visualized, providing the opportunity to independently ablate these subtypes and measure the effect on adult behavior in an intact animal (Fig 3B). We selectively induced bilateral ablations of LHLK neurons or unilateral ablation of two control ALK neurons in immobilized third-instar larvae with a titanium sapphire multiphoton laser. Ablation of individual neurons could be visualized in larvae as a disruption of the GFP-labeled neuronal cell body (Fig 3C). Following ablation, larvae were transferred back into food vials, and 5- to 7-day–old adult flies were tested for sleep under fed and starved conditions. After behavioral testing, brains were dissected and imaged to verify selective bilateral ablation of the LHLK neurons or unilateral ablation of two ALK neurons (Fig 3F). Flies with ablated ALK neurons suppressed sleep during starvation similarly to controls (Fig 3D and 3E). Conversely, bilateral ablation of the LHLK neurons abolished starvation-induced sleep suppression without affecting sleep while fed, revealing an essential role for the LHLK neurons in the integration of sleep and metabolic state (Fig 3D, 3E and 3F).

**Fig 4:**
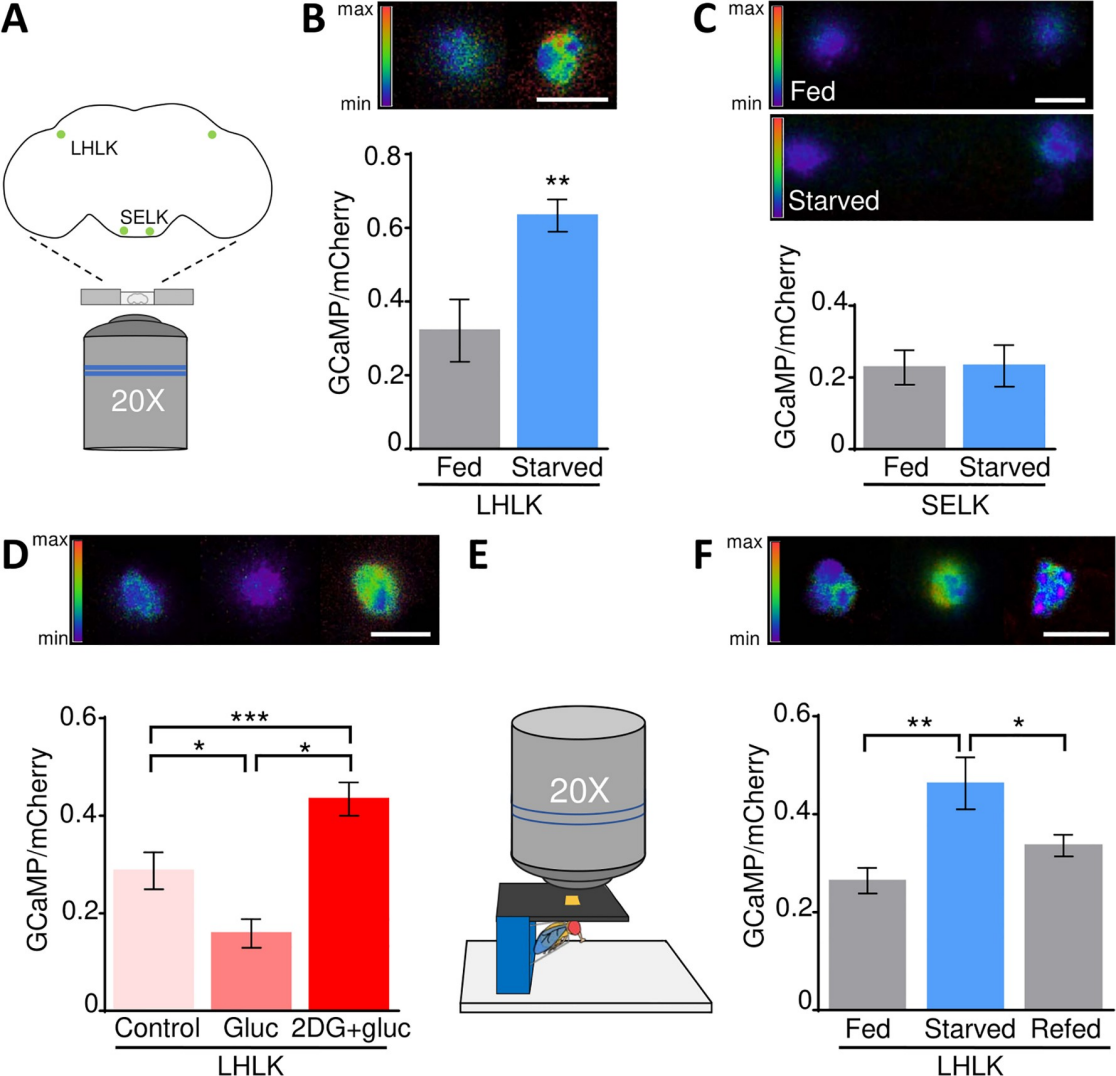
LHLK neurons have increased activity during the starved state. (A) Diagram of ex vivo Ca^2+^ imaging. Fed or 24-hr–starved adult female flies were dissected and placed dorsally onto chamber. UAS-Gerry fluorescence is recorded for 120 seconds with a Ti-Inverted Confocal microscope using a 20× air objective. (B) Average ratio of GCaMP/mCherry is increased in LHLK neurons during the starved state compared to the fed state ex vivo (*n* ≥ 8, *p* = 0.006, *t* = 3.154). Unpaired *t* test. (C) No significant differences in GCaMP/mCherry were detected in SELK neurons during the fed or starved state ex vivo (*n* ≥ 7, *p* = 0.95, *t* = 0.06). Unpaired *t* test. (D) Ex vivo application of 400 mM 2DG and 200 mM glucose to fed fly brains (*n* = 12) increases the GCaMP/mCherry fluorescence ratio in LHLK neurons compared to control (artificial hemolymph solution alone, *n* = 11, *p* = 0.01) and gluc application (200 mM, *n* = 12, *p* < 0.0001). Gluc application alone reduced the GCaMP/mCherry ratio compared to hemolymph-like solution control (*p* = 0.03). One-way ANOVA (F [2, 32] = 17.10). (E) Diagram of in vivo Ca^2+^ imaging. A portion of the head cuticle of a fed or 24-hr–starved adult female fly was removed, and GCaMP6m/mCherry fluorescence was recorded for 120 seconds. Fluorescence intensity scale represents the ratio range of GCaMP/mCherry ranging from 2 (max) to 0 (min). (F) GCaMP/mCherry ratio is increased in LHLK neurons during starvation (*n* = 30) compared to fed (*n* ≥ 19, *p* = 0.0036) and 3-hr re-fed controls (*n* = 29, *p* = 0.047). One-way ANOVA (F [2, 75] = 6.2). Scale bar = 10 μm. Error bars for GCamMP6m/mCherry ratio during the fed versus starved state indicate SEM; *p < 0.05; **p < 0.01; ***p < 0.001. Underlying data can be found in S1 Data. ANOVA, analysis of variance; GCaMP6m, GFP-calmodulin and M13 peptide sequence; GFP, green fluorescent protein; gluc, glucose; LHLK, Lateral Horn leucokinin; max, maximum; min, minimum; SELK, subesophageal ganglion leucokinin; UAS, upstream activation sequence; UAS-Gerry, GCaMP6m-mCherry; 2DG, 2 deoxy-glucose.

The finding that LHLK neurons are required for starvation-induced sleep suppression raises the possibility that the activity of Lk neurons is modulated by nutritional state. We selectively expressed a GFP-calmodulin and MP13 peptide sequence (GCaMP6m)-mCherry (UAS-Gerry) fusion protein that allows for ratiometric detection of Ca^2+^ activity [36,37] in Lk neurons and measured the response to nutrients. The brains of fed or 24-hr–starved flies were imaged for GCaMP and mCherry signal ex vivo (Fig 4A). Flies expressing Gerry in Lk neurons suppressed sleep similarly to control flies harboring Lk-GAL4 alone, indicating that expression of the Ca^2+^ sensor does not affect starvation-induced regulation of sleep (S3A Fig). The GCaMP/mCherry ratio was elevated in the LHLK neurons of starved flies compared to fed controls, suggesting these neurons are more active during starvation (Fig 4B). Conversely, no difference in the GCaMP/mCherry ratio between the fed and starved states was detected in the SELK neurons (Fig 4C).

In mammals, the activity of some sleep- and wake-promoting neurons are directly modulated by glucose and other circulating nutrients [38,39]. It is possible that the activity of Lk neurons is modulated in accordance with feeding state by sensory detection of tastants or indirectly result upon detection of changes in circulating nutrients. To differentiate between these possibilities, the brains of fed flies were removed and treated with either glucose or the competitive inhibitor of glycolysis, 2 deoxy-glucose (2DG) [40–42]. Application of glucose reduced Ca^2+^ activity in LHLK neurons compared to controls treated with *Drosophila* artificial hemolymph alone, suggesting these neurons are sensitive to circulating glucose (Fig 4D). To verify that application of glucose was specific to LHLK neurons, we applied glucose or artificial hemolymph while measuring Ca^2+^ response in SELK neurons. No significant differences in Ca^2+^ activity were found between controls and glucose application (S3B Fig). Further, the combined application of 2DG and glucose increased Ca^2+^ activity to levels greater than hemolymph alone, mimicking the starved state (Fig 4D). Taken together, these findings indicate that the activity of Lk neurons are modulated in accordance with nutrient availability and support the notion that the LHLK neurons are more active during starvation, thereby suppressing sleep.

To examine whether the activity in Lk neurons is modulated by feeding state in an intact animal, we performed in vivo recordings in tethered flies (Fig 4E). Briefly, a portion of the head capsule was removed so that the LHLK neurons were accessible. The activity of LHLK neurons was then recorded in flies that had been previously fed or starved for 24 hr. In agreement with ex vivo findings, the GCaMP/mCherry ratio was elevated in the LHLK neurons of starved flies, fortifying the notion that Lk neurons are more active during starvation (Fig 4F). Refeeding flies with standard food reduced the GCaMP/mCherry ratio 3 hr after refeeding (Fig 4F). Taken together, these findings suggest the activity of LHLK neurons are modulated by circulating nutrient levels.

**Fig 5:**
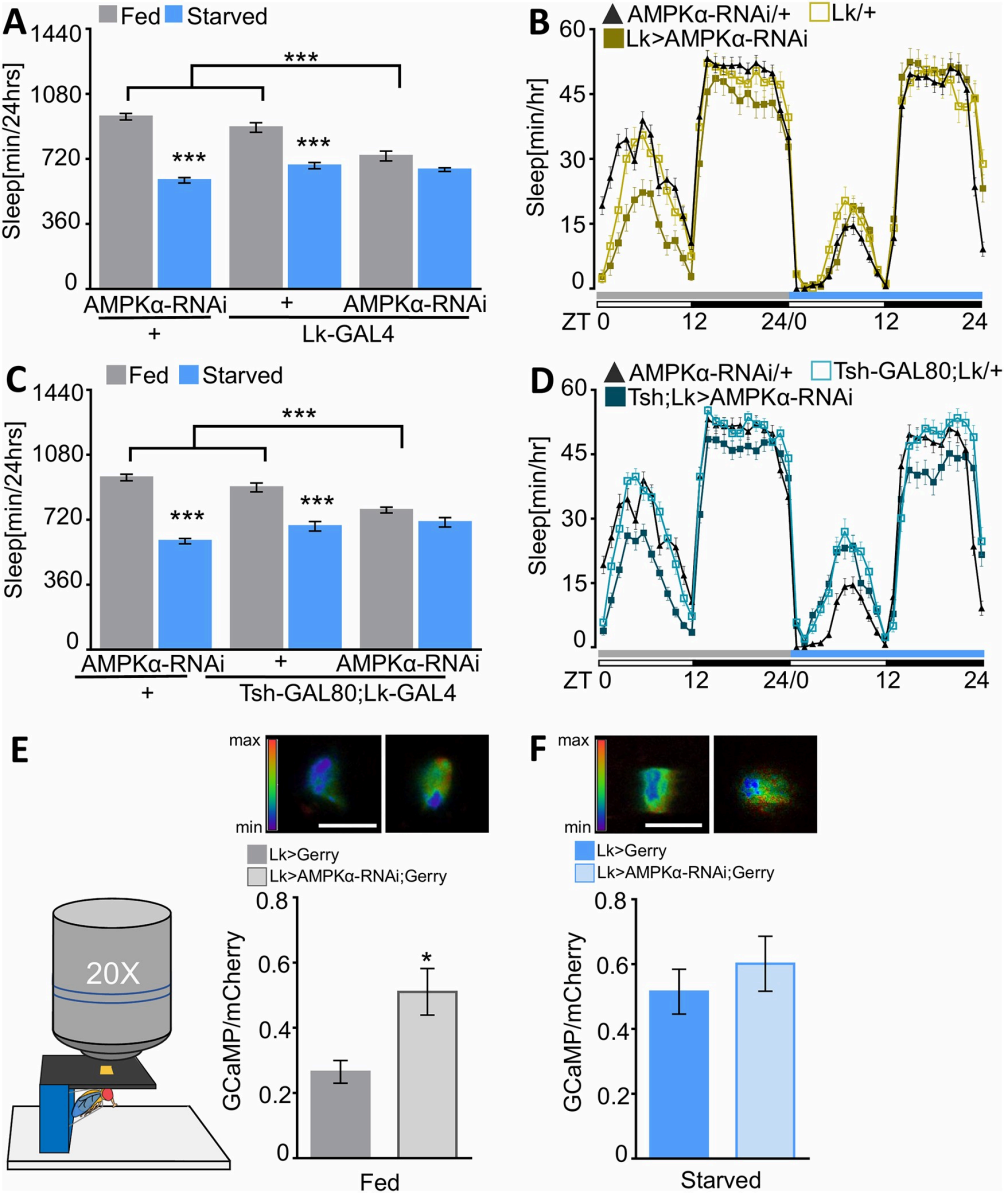
AMPKα in LHLK neurons is required for starvation-induced sleep suppression. (A) Knockdown of AMPKα in Lk neurons (Lk-GAL4, *n* = 32, *p* = 0.14) abolishes starvation-induced sleep suppression, while control flies Lk-GAL4/+ (*n* = 27, *p* < 0.0001) and AMPKα-RNAi/+ (*n* ≥ 58, *p* < 0.0001) suppress sleep. During the fed state, sleep is significantly reduced in Lk-GAL4>UAS-AMPKα-RNAi compared to controls Lk-GAL4/+ (*p* < 0.0001) and AMPKα-RNAi/+ (*p* < 0.0001). Two-way ANOVA (F [2, 230] = 22.70). (B) Sleep profile representative of (A). Flies are placed in food tubes during day 1 (fed, gray), then transferred to agar during day 2 (starved, blue). White/black bars represent lights on and off, respectively. (C) Flies expressing UAS-AMPKα-RNAi in brain Lk neurons (tsh-GAL80;Lk-GAL4) fail to suppress sleep in response to starvation (*n* ≥ 56, *p* = 0.16) compared to control flies that suppress sleep (tsh-GAL80;Lk-GAL4/+, *n* ≥ 45, *p* < 0.0001) and AMPKα-RNAi/+ (*n* ≥ 58, *p* < 0.0001). Sleep while fed is significantly reduced in tsh-GAL80;Lk-GAL4>UAS-AMPKα-RNAi compared to tsh-GAL80;Lk-GAL4/+ (*p* = 0.0004) and AMPKα-RNAi/+ (*p* < 0.0001). Two-way ANOVA (F [2, 364] = 23.12). (D) Sleep profile representative of (C). (E) In vivo Ca^2+^ imaging during the fed state shows an increase in the average ratio of GCaMP/mCherry in LHLK neurons expressing UAS-AMPKα-RNAi;UAS-Gerry (gray and white, *n* = 10) compared to flies harboring Gerry alone (gray; *n* = 8, *p* = 0.01, *t* = 2.86). Unpaired *t* test. (F) During the starved state, no significant differences in LHLK Ca^2+^ activity are observed in Lk-GAL4>UAS-AMPKα-RNAi;UAS-Gerry flies (blue and white, *n* = 8) compared to flies harboring Gerry alone (blue, *n* = 8, *p* = 0.44, *t* = 0.78), Unpaired *t* test. Fluorescence intensity scale represents the ratio range of GCaMP6m/mCherry ranging from 2 (max) to 0 (minimum). Scale bar = 10 μm. Error bars for GCamp6m/mCherry ratio during the fed versus starved state indicate SEM; *p < 0.05; **p < 0.01; ***p < 0.001. Underlying data can be found in S1 Data. AMPK, 5′ adenosine monophosphate-activated protein kinase; ANOVA, analysis of variance; GAL4, galactose-responsive transcription factor; GCaMP6m, GFP-calmodulin and M13 peptide sequence; GFP, green fluorescent protein; LHLK, Lateral Horn leucokinin; Lk, leucokinin; max, maximum; RNAi, RNA interference; tsh, teashirt; UAS, upstream activation sequence; UAS-Gerry, GCaMP6m-mCherry; ZT, Zeitgeber time.

The identification of nutrient-dependent changes in activity of LHLK neurons raises the possibility that cell-autonomous nutrient sensors or signaling pathways function within Lk neurons to modulate sleep. To identify regulators of sleep that modulate the activity of Lk neurons, we expressed RNAi targeted to 28 RNAi lines encoding putative nutrient sensors or signaling pathways using Lk-GAL4 and measured starvation-induced changes in sleep (S4A Fig). RNAi knockdown of AMP-activated protein kinase alpha (AMPKα) in Lk neurons (Lk-GAL4>UAS- AMPKα) alone abolished starvation-induced sleep suppression compared to GAL4 controls crossed to the isogenic host strain for the RNAi library (Fig 5A and 5B and S4A Fig) [43]. Feeding did not differ in flies expressing AMPKα-RNAi in Lk neurons, suggesting the sleep phenotype is not due to generalized changes in hunger (S4B Fig). Targeting AMPKα-RNAi with a second, independently derived RNAi line also abolished starvation-induced sleep suppression (Lk-GAL4>UAS-dcr2, UAS-AMPKα-RNAi #2; S4C Fig). Genetically restricting AMPK knockdown in flies harboring tsh-GAL80 (tsh-GAL80;Lk-GAL4>AMPKα-RNAi) also impaired starvation-induced sleep suppression (Fig 5C and 5D), suggesting AMPKα functions in the Lk neurons within the brain to regulate sleep.

To determine whether inhibition of AMPK signaling changes the activity of Lk neurons to resemble a starved-like state, we genetically expressed AMPKα-RNAi under control of Lk-GAL4 and measured neuronal activity in vivo using UAS-Gerry (Fig 5E and 5F; Lk-GAL4>UAS-AMPKα-RNAi; UAS-Gerry). Genetic disruption of AMPKα increased Ca^2+^ activity in LHLK neurons of fed flies compared to flies expressing UAS-Gerry alone (Fig 5E). The increase Ca^2+^ activity phenocopies changes found in starved control flies, suggesting that the loss of AMPK increases the activity of Lk neurons, thereby suppressing sleep (Fig 5F). Together, these findings suggest AMPK is active within LHLK neurons during the fed state, and reduced AMPK signaling during starvation increases LHLK activity.

**Fig 6:**
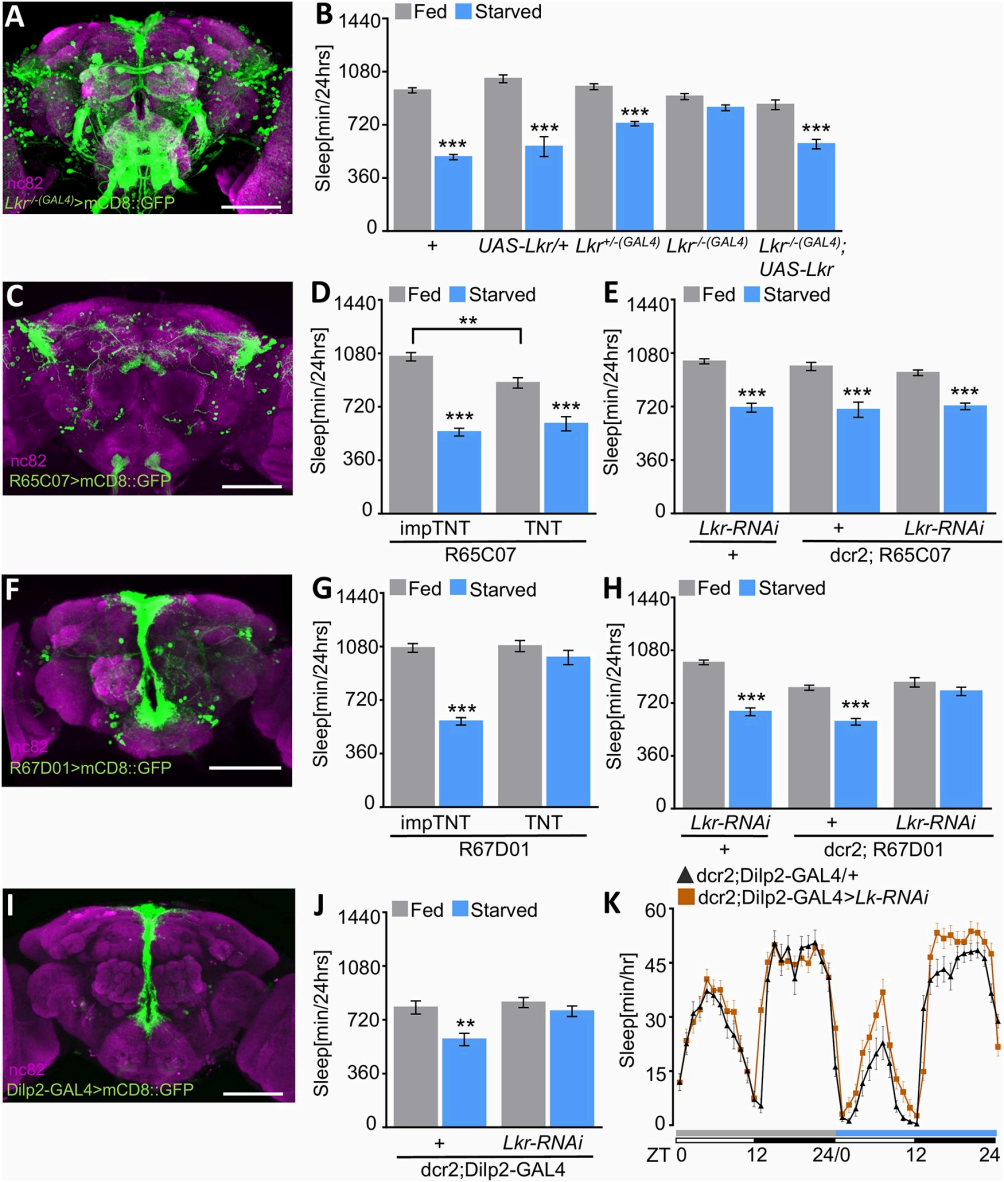
Lk targets the IPCs to promote wakefulness during starvation. Expression pattern for *Lkr*^−/−^>UAS-mCD8::GFP (A), R65C07-GAL4 (C), R67D01-GAL4 (F), and Dilp2-GAL4 (I). The brains were counterstained with nc82 (magenta). Scale bar = 100 μm. (B) *Lkr*^*−/−(GAL4)*^ mutants fail to suppress sleep in response to starvation (*n* ≥ 147, *p* = 0.09) compared to control *w*^*1118*^ flies (*n* = 155, *p* < 0.0001), *Lkr*^*+/−*^ (*n* ≥ 134, *p* < 0.0001), and *UAS-Lkr*/+ (*n* = 26, *p* < 0.0001). Expression of *UAS-Lkr* in neurons labeled by *Lkr*^*−/−(GAL4)*^ (*Lkr*^*−/−(GAL4)*^; *UAS-Lkr*) restores starvation-induced suppression (*n* = 28, *p =* 0.0003). There were no significant differences during the fed state between control (*UAS-Lk/+*) and rescue flies (*p* = 0.10) or *Lk*^−/−^ (*p* = 0.24). Two-way ANOVA (F [4, 965] = 13.05). (D) Blocking synaptic release in *Lkr*-expressing neurons that label the dFSB (R65C07-GAL4>UAS-TNT) does not affect starvation-induced sleep suppression (*n* = 48, *p* < 0.0001), similar to impTNT controls (*n* = 50, *p* < 0.0001). Sleep while fed is reduced in R65C07-GAL4>TNT flies compared to control (*p* = 0.0025). Two-way ANOVA (F [1, 109] = 11.17). (E) No impairments in starvation-induced sleep suppression were observed when knocking down *Lkr* in Lkr-expressing neurons (R65C07-GAL4>UAS-dcr2,*Lkr-RNAi*, *n* ≥ 45, *p* < 0.0001). Control *UAS-Lkr-RNAi*/+ (*n* = 27, *p* < 0.0001) and R65C07-GAL4/+ (*n* = 32, *p* < 0.0001) suppress sleep in response to starvation. Two-way ANOVA (F_column_ [1, 205] = 130.8). (G) Expression of TNT in R67D01-GAL4, which labels the IPCs, impairs starvation-induced sleep suppression (*n* = 31, *p* = 0.47), while impTNT control flies suppress sleep (*n* = 48, *p* < 0.0001). Two-way ANOVA (F [1, 154] = 37.02). (H) Starvation-induced sleep suppression is absent with *Lkr* knockdown in *Lkr*-expressing neurons (R67D01, *n* = 41, *p* = 0.53). Control *UAS-Lkr-RNAi*/+ (*n* = 25, *p* < 0.0001) and dcr2,R67D01-GAL4 (*n* ≥ 53, *p* < 0.0001) suppress sleep in response to starvation. Two-way ANOVA (F [2, 232] = 13.86). (J) Knocking down *Lkr* in the IPCs (Dilp2-GAL4>UAS-dcr2,*Lkr-RNAi*, *n* ≥ 30, *p* = 0.67) results in flies that fail to suppress sleep in response to starvation, while dcr2,Dilp2-GAL4/+ control flies suppress sleep (*n* = 25, *p* = 0.0016). Two-way ANOVA (F [1, 108] = 4.1). (K) Sleep profile representative of (J). Flies are placed in food tubes during day 1 (fed, gray), then transferred to agar during day 2 (starved, blue). White/black bars represent lights on and off, respectively. All columns are mean ± SEM; *p < 0.05; **p < 0.01; ***p < 0.001. Underlying data can be found in S1 Data. ANOVA, analysis of variance; CD8::GFP, membrane-tethered GFP; LK-GAL4>CD8:GFP;tshGAL80; dcr2, dicer-2; dFSB, dorsal fan-shaped body; Dilp2, *Drosophila* insulin-like peptide 2; GAL4, galactose-responsive transcription factor; GFP, green fluorescent protein; impTNT, inactive variant of tetanus toxin; IPC, insulin-producing cell; Lk, leucokinin; Lkr, leucokinin receptor; nc82, neuropil marker; RNAi, RNA interference; TNT, tetanus toxin; tsh, teashirt; UAS, upstream activation sequence; ZT, Zeitgeber time.

Lk signals through a single Lk receptor (*Lkr*) that is highly expressed in the IPCs and the dorsal fan-shaped body (dFSB), both of which have been implicated in sleep regulation [24,44–46]. To determine the role of *Lkr* in sleep regulation, we used CRISPR/Cas9 gene-editing to generate a recombinant transgenic line (*Lkr*^*−/−(GAL4)*^) with a GAL4 element inserted 106 to 111 base pairs preceding the ATG translational start site, disrupting its function. Consistent with previous reports of *Lkr* expression, transgene expression in *Lkr*^*−/−(GAL4)*^>UAS-mCD8::GFP flies labeled the IPCs, dFSB, and a number of other brain regions (Fig 6A). To determine the role of *Lkr* in sleep, we tested flies for sleep under fed and starved conditions. *Lkr*^*−/−(GAL4)*^ flies failed to suppress sleep when starved, phenocopying loss of *Lk* function (Fig 6B). Restoring *Lkr* to *Lkr* mutant flies (*Lkr*^*−/−(GAL4)*^>*UAS-Lk*r) rescued starvation-induced sleep suppression (Fig 6B). The promoter-fusion R65C07-GAL4 predominantly labels the dFSB, while R67D01-GAL4 primarily labels the IPCs ([47], Fig 6C and 6F). Silencing R65C07-GAL4–labeled neurons with TNT reduced sleep in fed flies, consistent with a sleep-promoting role for the dFSB ([45]; Fig 6D). However, there was no effect of *Lkr* knockdown in these cells (R65C07>UAS-dcr2, *Lkr-RNAi*), suggesting that Lk does not signal through the dFSB to modulate sleep. Conversely, genetic silencing or expression of *Lkr-RNAi* in R67D01-GAL4 neurons that label the IPCs abolished starvation-induced sleep suppression (Fig 6G and 6H). Together, these findings suggest *Lkr* function in the IPCs is required for starvation-induced sleep suppression. To validate a role for the IPCs, we selectively targeted *Lkr* in neurons labeled by *Drosophila* insulin-like peptide 2 (Dilp2) (Fig 6I). Selectively knocking down *Lkr* in Dilp2 neurons (Dilp2-GAL4>UAS-dcr2, *Lkr-RNAi*) prevented starvation-induced sleep loss, indicating that *Lkr* is required in *Dilp2* neurons for starvation-induced sleep suppression (Fig 6J and 6K).

## Discussion

Our findings reveal that Lk signals through a single Lkr to integrate sleep and metabolic state. Previous studies have identified a number of genes required for starvation-induced changes in sleep or locomotor activity, yet many of these genes have pleiotropic functions on behavior or metabolic function [20,29,48,49]. For example, the glucagon-like adipokinetic hormone (AKH) is responsible for energy mobilization, and genetic disruption of AKH induces obesity and abolishes starvation-induced hyperactivity [29,50,51]. Similarly, the circadian transcription factors *clock* and *cycle* are required for starvation-dependent regulation of behavior, and loss of function affects sleep both in fed and starved conditions [20]. Conversely, neuropeptide F functions within a subpopulation of circadian neurons and is selectively required for metabolic regulation of sleep [22]. Our findings that genetic manipulations that inhibit Lk signaling selectively disrupt starvation-induced modulation of sleep suggest that different neural mechanisms regulate sleep under basal conditions and in response to environmental perturbation.

The failure of *Lk* mutants to suppress sleep under starved conditions phenocopies mutation of the RNA/DNA binding protein trsn. Loss of trsn does not impact feeding behavior but impairs starvation-induced sleep suppression, suggesting that trsn is not generally required for hunger-induced behavior [23]. While *trsn* is broadly expressed in the fly nervous system [52,53], we previously found that selective knockdown of *trsn* in Lk neurons disrupted starvation-induced sleep suppression [54]. These findings raise the possibility that trsn functions to regulate changes in the physiology of Lk neurons to modulate sleep under starved conditions.

A central question is how Lk neurons modulate numerous complex behaviors and physiological processes. In adults, Lk is expressed in four pairs of neurons in the brain and 11 pairs in the ventral nerve cord, which regulate diverse behaviors and physiological processes [55–57]. Recent work suggests the ABLK neurons in the thoracic ganglion are critical regulators of water consumption and contribute to the altered stress resistance and water content in Lk mutant flies [46]. The SELK neurons connect the gustatory receptors to the subesophageal ganglia and ventral nerve cord. Although a specific function has not been identified to SELK neurons, silencing of all Lk neurons disrupts gustatory behavior, and a mutation in the *Lk* locus affects meal size [28,58], raising the possibility that these behaviors are regulated by SELK neurons. Lastly, the LHLK neurons project to the superior lateral protocerebrum, medial protocerebrum, and peduncle and axonal stalk of the mushroom bodies [56]. The LHLK neurons we identify here as regulating sleep–metabolism interactions receive inputs from clock neurons and are thought to modulate locomotor activity and sleep [27]. Supporting the notion that LHLK neurons are outputs of the clock, previous work has found that silencing Lk within these neurons attenuates circadian rhythms [27]. Conversely, we find that silencing of these neurons has little effect on sleep yet abolishes starvation-induced sleep suppression. Together, these findings support a role for LHLK neurons in sleep regulation, yet discrepancies remain about the conditions under which these neurons regulate sleep.

Lk neurons have also been implicated in the modulation of sleep following the ingestion of a meal. Postprandial sleep is enhanced in Lk-deficient flies and reduced in flies with thermogenetically activated Lk neurons sleep, whereas Lk knockdown increases the probability of falling asleep after a meal [59]. Similarly, we found that knockdown of Lk in Lk-expressing neurons results in increased sleep during starvation. In contrast, it was previously reported that Lk signals the fan-shaped body neurons to regulate postprandial sleep [59]. These results raise the possibility that distinct circuitry regulating starvation-induced sleep suppression and postprandial sleep, which could be influenced by distinct time-scales at which both behaviors are being executed. In addition, it is possible that a distinct subpopulation of Lk neurons is responsive to individual nutrients in comparison to starvation itself, which is sensed by LHLK neurons. Taken together, these findings suggest different neural mechanisms underlie Lk-dependent regulation of postprandial feeding and circadian rhythms.

The *Drosophila* genome encodes for a single Lk target, the *Lkr*, that is expressed in the lateral horn, the ventral nerve cord, the IPCs, and the sleep-promoting fan-shaped body [27,28,59]. The fan-shaped body, a subregion of the *Drosophila* central complex, is a primary sleep-promoting region [45,60], while the IPCs are proposed integrators of sleep and feeding state [61]. Previous studies suggest that *Lkr* function within the fan-shaped body is required for proper regulation of circadian rhythms and postprandial sleep [27,59]. Here, we find that targeted knockdown of *Lkr* within the IPCs phenocopies LHLK ablation, suggesting the LHLK neurons signal to the IPCs to modulate starvation-induced regulation of sleep. Supporting these findings, recent work has shown that *Lk* and *Lkr* mutants display increased levels of Dilp2 and Dilp3 immunoreactivity in the IPCs [46]. However, anterograde trans-synaptic labeling revealed no direct synaptic inputs between Lk neurons and the IPCs, raising the possibility that that Lk inputs to the IPCs could occur via paracrine signaling [46]. Taken together with previous studies, these findings suggest different Lk targets regulate postprandial feeding, circadian rhythms, and sleep or that Lk functions through paracrine signaling to modulate different targets.

In *Drosophila*, a number of circulating nutrients including fructose, trehalose, and glucose have been found to affect central brain physiology and behavior [42,62,63]. While nutrients may be detected by gustatory receptors expressed in the periphery to regulate sleep [9,64], sugar receptors and transporters are also expressed in the brain [65]. The identification of LHLK neurons as being active under starvation conditions and suppressed by glucose provide a system to investigate feeding-state–dependent changes in neural activity. A number of neurons in the fly brain are acutely regulated by feeding state, including the starvation-active Taotie neurons that inhibit IPCs of the pars intercerebralis to regulate insulin-like peptide release under nutrient deprivation conditions [66–68]. Conversely, the IPCs function as cell-autonomous nutrient sensors that are activated by glucose through the inhibition of K_ATP_ channels [69]. Further, the LHLK nutrient phenotype is similar to the neurons within the ellipsoid body labeled by the sodium/glucose co-transporter SLC5A11 that are active during starvation and promote feeding [41,70]. SLC5A11 and its cognate neurons are required for a variety of hunger-induced feeding behaviors, but the effect on sleep has not been identified [70]. Our screen found that knockdown of SLC5A11 in Lk neurons did not affect starvation-induced sleep suppression, suggesting alternative regulators of sleep. The identification of LHLK neurons as starvation-active neurons provides a system for identification of additional nutrient sensors that regulate sleep.

AMPK functions as a cell-autonomous regulator of energy allocation and induces physiological changes associated with starvation [71,72]. AMPK consists of a heterotrimeric complex that is activated by AMP and modulates diverse intercellular signaling pathways, including mammalian target of rapamycin (mTOR), forkhead box (FoxO), and sirtuin-1 (SIRT1) [73]. Canonically, AMPK is activated during starvation and increases neuronal activity, though this effect varies by neuronal subtype [74,75]. For example, in *Caenorhabditis elegans*, starvation-induced AMPK activation leads to inhibition of neurons that modulate local search behavior in response to food deprivation while promoting activity in neurons that trigger dispersal behavior [76]. Here, we find that knockdown in LHLK neurons using multiple independently derived RNAi lines results in flies that reduce sleep under the fed state and increases the activity of LHLK neurons, similar to neural activity seen during the starved state. Ubiquitous disruption of AMPK in *Drosophila* induces hypersensitivity of the locomotor response to starvation and reduces starvation resistance [74]. Conversely, we find that selectively disrupting AMPK function in Lk neurons promotes starvation-induced hyperactivity and sleep loss during the fed state, suggesting a neural-circuit–specific function for AMPK. While our findings suggest that AMPK functions as an important modulator of LHLK neuronal activity and state-dependent changes of activity within LHLK neurons, it is also possible that AMPK generally modulates the activity of Lk neurons, resulting in sleep loss. The findings that the activity of SELK neurons is not elevated during starvation raises the possibility of neuron-specific AMPK function.

Taken together, the identification of LHLK neurons as critical modulators of sleep–metabolism interactions provides a system for identifying novel nutrient-sensing and signaling mechanisms that modulate sleep. These findings illustrate the need to determine how Lk neurons modulate different aspects of sleep regulation, including their reported role as circadian output neurons, in regulation of sleep–metabolism interactions, and in postprandial sleep regulation [23,27,59]. Further investigation of feeding-state–dependent changes in Lk signaling and the identification of neuronal inputs and targets of LHLK neurons will provide mechanistic insight into how animals integrate sleep with changes in their internal and external environments.

## Materials and methods

### *Drosophila* maintenance and fly stocks

Flies were grown and maintained on standard food (Bloomington Recipe, Genesee Scientific, San Diego, CA, USA). Flies were kept in incubators (Dros52; Powers Scientific, Warminster, PA, USA) at 25 °C on a 12:12 LD cycle with humidity set to 55%–65%. The background control line used in this study is the *w*^*1118*^ fly strain, and all experimental flies were outcrossed 6–8 generations into this background. All the experiments performed in this manuscript used mated female flies. The following fly strains were ordered from Bloomington Stock Center: *w*^*1118*^ (5905; [77]), Lk^c275^ (16324; [28]), elav-GAL4 (8765; [78]), Apt-GAL4 (3041; [79]), UAS-TNT (28996; [31]), UAS-impTNT (28840; [31]), UAS-mCD8::GFP (32186; [80]), UAS-dcr2 (Chr II;24650; [43]), UAS-dcr2 (Chr III;24651; [43]), AMPKα-RNAi#2 (35137; [81]), *UAS-Lkr-RNAi* (65934; [81]), UAS-luciferase (31603; [81]), Lkr-GAL4 (39344; [47]), and Lkr-GAL4 (39412; [47]). The following lines were generated in this study: *Lk*^*−/−(GAL4)*^, *Lkr*^*−/−GAL4)*^, and *UAS-Lk*. UAS-Gerry was a kind gift from Greg Macleod, Lk-GAL4 and Dilp2-GAL4 from Young-Joon Kim, and *UAS-Lk*r from Bader Al Anzi [28]. tsh-GAL80 [30] was provided by Julie Simpson. *Drosophila* lines used in the RNAi screen and *UAS-Lk-RNAi* (14091) originate from the VDRC library [43] and are described in Table 1.

**Table 1:** Fly strains used in the screen.

Name	CG	Vienna Stock center ID	FlyBase ID
GD control		V6000	
cupcake	CG8451	V48987(1) v3424	FBgn0031998
npfr1	CG1147	v9605	FBgn0037408
Gr43a	CG1712	v39518	FBgn0041243
Glut 1	CG43946	v47179(2) v47178(3) v13326(1)	FBgn0264574
Sut-1	CG5772	v9950	FBgn0028563
Tret-1	CG30035	v52360(3) v52361(2) v8126(1)	FBgn0050035
TASK-6	CG9637	v9073	FBgn0038165
TASK-7	CG9361	v8565	FBgn0037690
dIRK	CG44159	v28430(1) v28431(2)	FBgn0039060
dIRK-2	CG4370	v4341	FBgn0039081
dIRK-3	CG10369	v3886	FBgn0032706
dSUR	CG5772	v6750	FBgn0025710
Hex-C	CG8094	v35337	FBgn0001187
AMPKα	CG3051	v1827	FBgn0023169
PKA C2	CG12066	v30658(1) v30685(2)	FBgn0000274
PKA R1	CG42341	v26328	FBgn0000275
PKA R2	CG15862	v39437(1) v39436(2)	FBgn0000353
Prestin	CG5485	v5341	FBgn0036770
MCT	CG8028	v9163	FBgn0031010
Silnoon	CG8271	v4607	FBgn0033657

**Abbreviations**: AMPK, 5′ adenosine monophosphate-activated protein kinase.

### Generation of GAL4 knock-in mutants and *UAS-Lk*

*Lk*^*−/−(GAL4)*^ and *Lkr*^*−/−(GAL4)*^ were generated by Wellgenetics (Taipei City, Taiwan) using the CRISPR/Cas9 system to induced homology-dependent repair (HDR) using one guide RNA (gRNA) (*Lk*^*−/−(GAL4)*^: GATCTTTGCCATCTTCTCCAG and *Lkr*^*−/−(GAL4)*^: GTAGTGCAATACATCTTCAG). At the gRNA target site, a donor plasmid was inserted containing a GAL4::VP16 and floxed 3xP3-RFP cassette [82]. For *Lk*^*−/−(GAL4)*^, following the translational ATG start site, bases 1 to 7 were replaced by the knock-in cassette. For *Lkr*^*−/−(GAL4)*^, preceding the ATG start site, bases 111 to 106 were replaced by the knock-in cassette. All lines were generated in the *w*^*1118*^ background [77]. Proper insertion loci for both mutations were validated by genomic PCR.

### *UAS*-*Lk*

The full-length open reading frame of Lk was amplified from the Lk-pOT2 plasmid (Drosophila Genomics Resource Center [DGRC], #1378621) using specific primers (forward primer: GCCTTTGGCCGTCAAGTCTA and reverse primer: CTCCAAGTACCGCAGGTTCA) generated by Integrated DNA Technologies (Coralville, IA, USA). Amplified sequence was inserted into the pENTER vector (Invitrogen) via TOPO cloning and subsequently recombined into pTW destination vector (DGRC, #1129) using standard gateway cloning protocol as per manufacturer’s instructions (Invitrogen, Carlsbad, CA, USA). The plasmids were verified by sequencing (Genewiz, Morrisville, NC, USA). Transgenic lines were established via phiC31-mediated integration at the attp40 landing site [83] on the second chromosome (BestGene, Chino Hills, CA, USA).

### Behavioral analysis

The DAMS detects activity by monitoring infrared beam crossings for each animal [84]. These data were used to calculate sleep information by extracting immobility bouts of 5 minutes using the *Drosophila* Counting Macro [85,86]. For experiments examining the effects of starvation on sleep, flies were kept on a 12:12 LD cycle. Mated female flies were briefly anesthetized with CO_2_ and placed into plastic tubes containing standard food. All flies were given 24 hr to recover after being anesthetized. Activity was recorded for 24 hr in food tubes prior to transferring flies into tubes containing 1% agar diluted in dH_2_O (Fisher Scientific) at Zeitgeber time (ZT) 0. Activity was monitored for an additional 24 hr on agar. For the screen, percent change in sleep during starvation was calculated as the sleep duration on agar minus the sleep duration in food tubes, divided by the sleep duration in food tubes for each fly assayed multiplied by a hundred [11,54].

### Immunohistochemistry

The brains of 5- to 7-day–old female flies were dissected between ZT 4–ZT 9 in ice-cold phosphate-buffered saline (PBS) and fixed in 4% paraformaldehyde, PBS, 0.5% Triton-X for 30 minutes as previously described [87]. Brains were then rinsed 3× with PBS, 0.5% Triton-X (PBST) for 10 minutes and overnight. In the following day, brains were incubated for 24 hr in primary antibody (1:1,000 rabbit anti-Lk [88] and mouse 1:20 nc82; Iowa Hybridoma Bank, University of Iowa, Iowa City, IA, USA) diluted in PBST at 4 °C. Brains were rinsed in PBST 3× for 10 minutes and placed in secondary antibody (1:400 donkey anti-rabbit Alexa 555 and 1:200 donkey anti-mouse Alexa 647; Thermo Fisher Scientific, Waltham, MA, USA), diluted in PBST for 90 minutes at room temperature. Finally, all samples were washed in PBST for a total of 120 minutes and mounted in Vectashield (VectorLabs, Burlingame, CA, USA). Samples were imaged in 2-μm sections with a Nikon A1R confocal microscope (Nikon, Tokyo, Japan) using a 20× or 60× oil immersion objective. Images were then processed with NIS Elements 4.40 (Nikon).

### Blue-dye assay

Briefly, flies were maintained on standard fly food. At ZT 0, flies were transferred to vials containing 1% agar, 5% sucrose, and 2.5% blue dye (FD&C Blue Dye No. 1). Following 30 minutes of feeding, flies were flash frozen on dry ice, and four flies were homogenized in 400 μL PBS (pH 7.4, Thermo Fisher Scientific) per sample. Color spectrophotometry was then used to measure absorbance at 655 nm in a 96-well plate reader (iMark; Millipore Sigma, Burlington, MA, USA). Baseline absorbance was determined by subtracting the absorbance measured in non-dye–fed flies from each experimental sample.

### Functional imaging of Lk neurons

Five- to seven-day–old female flies were collected and placed in vials containing fresh food (fed) or a wet KimWipe paper (starved) for 24 hr. All experiments were done between ZT 4–ZT 7 to account for rhythmic excitability of Lk neurons [27]. For imaging brain explants, previously established methods for calcium imaging were used with modifications [42,65]. Brains of fed or 24-hr–starved flies were dissected and placed in glass wells (Pyrex, Corning, Corning, NY, USA) containing artificial hemolymph (140 mM NaCL, 2 mM KCl, 4.5 mM MgCl2, 1.5 mM CaC2, and 5 mM HEPES-NaOH with pH 7) and allowed a 5-minute recovery period before being recorded. For 2DG experiments, fed brains were dissected and placed in 400 mM 2DG (Sigma Aldrich) in artificial hemolymph, 200 mM glucose (Sigma Aldrich) in artificial hemolymph, or artificial hemolymph alone for a total of 70 minutes. Every 20 minutes, solutions were exchanged. Coverslips were treated with poly-L-lysine (Sigma Aldrich) to ensure that brains were in the same position during imaging and placed onto chamber (RC-21BBDW; Warner Instruments, Hamden, CT, USA). Fly brains were bathed in artificial hemolymph solution and imaged using a 20× air objective lens on an inverted confocal microscope (Nikon A1R on a Ti-E inverted microscope). The pinhole was opened to 244.43 μm to allow a thicker optical section to be monitored. UAS-GCaMP-R (GCaMP and mCherry) was expressed in Lk neurons and simultaneously excited with wavelengths of 488 nm (FITC) and 561 nm (TRITC). Recording were taken for 120 seconds, capturing 1 frame/5 seconds with 512 × 512 resolution. For analysis, regions of interest (ROIs) were drawn manually, capturing the same area between experimental and control. The mean fluorescence intensity was subtracted from background mean fluorescence intensity for FITC and TRITC per frame. Then, the ratio of GCaMP6.0 to mCherry was calculated and plotted as an average of the total time recorded per brain imaged.

In vivo imaging was performed using a previously described protocol with some modifications [89,90]. Briefly, fed, 24-hr–starved, or 3-hr re-fed (standard Bloomington Recipe) flies were anesthetized on ice and secured in a 200-μL pipette tip with head and proboscis accessible. The pipette tip was placed in a small chamber at an angle of 140°, allowing the dorsal and posterior surface of the brain to be imaged. A small hole was cut in the tin foil and fixed to the stage and fly head, leaving a window of cuticle exposed, then sealed using dental glue (Tetric EvoFlow; Ivoclar Vivadent, Schaan, Lichtenstein). The proboscis was extended, and dental glue was used to secure it in place, ensuring the same position throughout the experiment.

A 21-gauge 1 1/4 needle (PrecisionGlide; Becton Dickinson, Franklin Lakes, NJ, USA) was used to cut a window in the fly cuticle. A drop of artificial hemolymph was placed on the cuticle, and the connective tissue surrounding the brain was dissected. Flies were allowed to recover from the procedure for 30–45 minutes in a humidified box. Mounted flies were placed under a confocal microscope (Nikon A1R on an upright microscope) and imaged using a 20× water-dipping objective lens. The pinhole was opened to 244 μm to allow a thicker optical section to be monitored. The settings and data analysis were performed as described above.

### Targeted multiphoton ablation of Lk neurons

Female third-instar larvae expressing GFP in Lk neurons were selected and anesthetized in ethyl ether (Thermo Fisher Scientific, E134-1) for 2–5 minutes. Larvae were placed dorsally on a microscope slide, and a coverslip was placed on the larvae. Ringer’s solution was applied onto the larvae below the coverslip. Larvae were imaged using a 25× water-dipping objective lens on a multiphoton microscope (Nikon A1R) containing a Chameleon Vision II Ti:Sapphire tunable laser. Excitation laser light of 870 nm was used. Images were acquired at 1 frame per second with a resolution of 512 × 512 pixels. For each neural ablation, a total of four frames were acquired. Two frames were captured prior to ablation for a duration of approximately 2 seconds, followed by ROI stimulation of 2–4 seconds and two frames after ablation. Following ablations, larvae were placed in vials containing food and allowed to grow. Sleep in food tubes and on agar was measured 5–7 days posteclosion in the DAMS. In order to verify which neurons were ablated after behavioral assay, flies were anesthetized on ice, and the central nervous system (CNS) was dissected. Fly CNS was fixed in 4% paraformaldehyde, 0.5% PBST for 30 minutes. Following fixation, samples were imaged in 2-μm sections with a Nikon A1R confocal microscope (Nikon) using a 20× oil immersion objective. Ablations that resulted in the formation of supernumerary neurons or deletions of two different subpopulations of Lk neurons were removed from analysis.

### Statistical analysis

The experimental data are presented as means ± SEM. Unless otherwise noted, a one-way or two-way analysis of variance (ANOVA) followed by Tukey’s post hoc test was used for comparisons between two or more genotypes and one treatment and two or more genotypes and two treatments. Unpaired *t* test was used for comparisons between two genotypes. All statistical analyses were performed using InStat software (GraphPad Software 6.0) with a 95% confidence limit (*p* < 0.05).

## Supporting information

S1 Fig Lk neuropeptide is required for metabolic regulation of sleep. (A) The genomic organization of the *Lk* locus. *Lk*^*c275*^ consists of a piggyBac element inserted 929 base pairs 5′ to the transcription start site of the *leucokinin* gene (gold triangle). The dotted line corresponds to the cleavage site used for *Lk*^*−/−(GAL4)*^ mutant generation by CRISPR/Cas9-mediated Lk genome engineering. *Lk*^*−/−(GAL4)*^ contains a GAL4 element replacing base 1 to 7 downstream of the ATG site and a floxed 3xP3-RFP cassette (brackets, pale orange and turquoise). (B) Quantification of Lk peptide levels in LHLK neurons of dcr2,*Lk-RNAi*, Lk^c275^, and *Lk*^*−/−(GAL4)*^ mutants. (C) Waking activity in *Lk*^*c275*^ mutants does not differ between the fed and starved states (*n* = 59, *p* > 0.99). Control flies (*w*^*1118*^, *n* = 64, *p* < 0.0001) and *Lk*^*c275*^/+ (*n* = 66, *p* = 0.0015) increase waking activity during starvation. Two-way ANOVA (F [2, 374] = 29.07). (D) Control flies (*w*^*1118*^, *n* = 77, *p* < 0.0001) and *Lk*^*+/−(GAL4)*^ (*n* = 70, *p* = 0.0001) increase waking activity during starvation, while waking activity does not differ between the fed and starved states in *Lk*^*−/−(GAL4)*^ (*n* = 47, *p* = 0.61). Two-way ANOVA (F [2, 382] = 17.47). (E) Pan-neuronal rescue of *Lk*^*c275*^ (elav-GAL4;*Lk*^*c275*^>*UAS-Lk*;*Lk*^*c275*^, *n* = 17, *p* = 0.04) restores starvation-induced sleep suppression compared to *Lk*^*c275*^ mutant controls *UAS-Lk/+*;*Lk*^*c275*^ (*n* = 24; *p* > 0.99) and elav-GAL4/+;*Lk*^*c275*^ (*n* ≥ 20, *p* = 0.99). Sleep duration on agar (starved) does not differ significantly between rescue and *UAS-Lk/+*;*Lk*^*c275*^/+ (*n* = 30, *p* = 0.08) or elav-GAL4/+;*Lk*^*c275*^/+ (*n* = 51, *p* = 0.11). Two-way ANOVA (F [4, 272] = 8.97). White bars in column graphs represent amount of sleep during the day (ZT 0–12), while colored bars represent night sleep (ZT 12–24). (F) Pan-neuronal rescue of *Lk*^*c275*^ (elav-GAL4;*Lk*^*c275*^>*UAS-Lk*;*Lk*^*c275*^) (*n* = 17, *p* = 0.02) restores starvation-induced increase in waking activity compared to *Lk*^*c275*^ mutant controls *UAS-Lk/+*;*Lk*^*c275*^ (*n* = 23, *p* = 0.37) and elav-GAL4/+;*Lk*^*c275*^ (*n* = 20, *p* > 0.99). No significant differences were seen during the starved state between control flies *UAS-Lk/+*;*Lk*^*c275*^/+ (*n* = 30, *p* > 0.94) or elav-GAL4/+;*Lk*^*c275*^/+ (*n* = 51, *p* > 0.99) and rescue flies. Two-way ANOVA (F [4, 272] = 2.93). (G) Whole-brain confocal reconstruction of *Lk*^*−/−(GAL4)*^>mCD8:GFP. The brain was counterstained with neuropil marker (nc82) (magenta). Scale bar = 100 μm. (H) Expression of *UAS-Lk* in neurons labeled by *Lk*^*−/−(GAL4)*^ restores starvation-induced suppression (*n* ≥ 27, *p* = 0.001) compared to *Lk*^*−/−(GAL4)*^ flies (*n* = 15, *p* = 0.97). Flies harboring one copy of the *UAS-Lk* alone (*UAS-Lk*/+) suppress sleep in response to starvation (*n* = 52, *p* < 0.0001). There were no significant differences during the fed state between control (*UAS-Lk/+*) and rescue flies (*p* = 0.91) or *Lk*^*−/−(GAL4)*^ (*p* = 0.95). Two-way ANOVA (F [2, 185] = 5.32). (I) Increase in waking activity following starvation is recued in flies expressing *UAS-Lk* under control of *Lk*^*−/−GAL4)*^ (*n* = 30, *p* = 0.03) compared to *Lk*^*−/−(GAL4)*^ mutants (*n* = 15, *p* > 0.99). Flies harboring *UAS-Lk* alone (*UAS-Lk*/+) increased waking activity following starvation (*n* = 50, *p* < 0.0001). There were no significant differences during the fed state between *UAS-Lk*/+ and rescue (*p* > 0.99) or *Lk*^*−/−(GAL4)*^ (*p* = 0.90). Two-way ANOVA (F [2, 184] = 3.81). (J) Genetic rescue (*UAS-Lk*;*Lk*^*+/−(GAL4)*^) (*n* = 81, *p* < 0.0001) restores starvation-induced suppression compared to flies harboring one copy of *Lk*^*+/−(GAL4)*^ (*n* = 68, *p* = 0.28). No significant differences were observed during the starved state between heterozygous rescue flies and control flies harboring a copy of *UAS-Lk* alone (*n* = 52, *p* = 0.79). Two-way ANOVA (F [2, 396] = 13.66). All columns are mean ± SEM; *p < 0.05; **p < 0.01; ***p < 0.001. Underlying data can be found in S1 Data. ANOVA, analysis of variance; CD8, XXX; CD8::GFP, LK-GAL4>CD8:GFP;tshGAL80; CRISPR/Cas9, Clustered Regularly Interspaced Short Palindromic Repeats; dcr2, dicer-2; elav, embryonic lethal abnormal vision; GAL4, galactose-responsive transcription factor; GFP, green fluorescent protein; LHLK, Lateral Horn leucokinin; Lk, leucokinin; mCD8::GFP, membrane-tethered GFP; nc82, neuropil marker; RFP, red fluorescent protein; RNAi, RNA interference; tsh, teashirt; UAS, upstream activation sequence; ZT, Zeitgeber time.
(TIF)

S2 Fig LHLK neurons are required for the metabolic regulation of sleep. (A) Expression pattern of Apt-GAL4 driving mCD8::GFP (green) and endogenous expression of Lk neuropeptide (red). The brain was counterstained with nc82 (gray). Scale bar = 50 μm. (B) Immunostaining for anti-LK (red) in Apt-GAL4>mCD8::GFP (green) reveals LHLK localizes to neurons labeled by Apt-GAL4 (orange, top panel). SELK neurons (bottom panel) do not colocalize with SOG neurons labeled by Apt-GAL4. Depicted is a 14-μm section from the lateral horn region and a 6-μm section from the SOG region using a 60× oil immersion objective. Scale bar = 10 μm. Apt, apterous; CD8::GFP, LK-GAL4>CD8:GFP;tshGAL80; GAL4, galactose-responsive transcription factor; GFP, green fluorescent protein; LHLK, Lateral Horn leucokinin; Lk, leucokinin; mCD8::GFP, membrane-tethered GFP; nc82, neuropil marker; SELK, subesophageal ganglion leucokinin; SOG, subesophageal ganglion; tsh, teashirt.
(TIFF)

S3 Fig LHLK neurons have increased activity during the starved state. (A) Flies expressing UAS-Gerry in Lk-GAL4 sleep significantly more in food tubes (gray) than on agar (blue, *n* = 24, *p* < 0.0001) similar to control flies, UAS-Gerry/+ (*n* = 32, *p* < 0.0001), Lk-GAL4/+ (*n* = 31, *p* < 0.0001), or *w*^*1118*^ flies (*n* = 32, *p* < 0.0001). No significant differences were detected in the fed state between Lk-GAL4>UAS-Gerry and *w*^*1118*^ control (*p* = 0.45), UAS-Gerry alone (*p* > 0.99), or Lk-GAL4 alone (*p* = 0.99). Two-way ANOVA, (F [3, 230] = 0.97). All columns represent the mean ± SEM; ***p < 0.001. (B) No significant differences in GCaMP/mCherry were detected in SELK neurons in controls bathed with artificial hemolymph solution alone or 200 mM of glucose (*n* ≥ 4, *p* = 0.77, *t* = 0.3). Unpaired *t* test. Scale bar = 10 μm. Fluorescence intensity scale represents the ratio range of GCaMP6m/mCherry ranging from 5 (max) to 0 (min). Underlying data can be found in S1 Data. ANOVA, analysis of variance; GAL4, galactose-responsive transcription factor; GCaMP6m, GFP-calmodulin and M13 peptide sequence; LHLK, Lateral Horn leucokinin; Lk, leucokinin; max, maximum; min, minimum; SELK, subesophageal ganglion leucokinin; UAS, upstream activation sequence; UAS-Gerry, GCaMP6m-mCherry.
(TIF)

S4 Fig AMPKα functions in the LHLK neurons to regulate sleep. (A) The percentage change in sleep in an RNAi screen targeting nutrient sensors or signaling pathway molecules in Lk-GAL4 neurons. Greater sleep suppression was observed in controls Lk-GAL4 and the isogenic host strain for the Vienna *Drosophila* Resource Center RNAi library (Lk-GAL4/+, *n* = 56) compared to AMPKα-RNAi (*n* = 9, *p* = 0.04). One-way ANOVA with Dunnett, F (28, 358) = 4.47). Dashed lines indicate control mean ± 2 SD. (B) Knockdown of AMPKα in Lk neurons (Lk-GAL4> AMPKα-RNAi, *n* = 9) does not affect food intake during the fed state compared to AMPKα-RNAi/+ (*n* = 13, *p* = 0.65) and Lk-GAL4/UAS-dcr2,*luc*-RNAi (*n* = 12, *p* = 0.92). One-way ANOVA F (2, 31) = 0.40. (C) Expression of a second AMPKα-RNAi line in Lk neurons (Lk-Gal4>UAS-dcr2,AMPKα-RNAi) abolishes starvation-induced sleep suppression (*n* = 45, *p* = 0.76), while control flies dcr2,Lk-GAL4/+ (*n* = 32, *p* < 0.0001) and AMPKα-RNAi/+ (*n* = 44, *p* = 0.002) suppress sleep. In fed flies, sleep is significantly reduced in Lk-GAL4>UAS-dcr2, AMPKα-RNAi compared to Lk-GAL4/UAS-dcr2,*luc*-RNAi (*p* = 0.0004) and AMPKα-RNAi/+ (*p* = 0.04) controls. Two-way ANOVA (F [2, 236] = 8.89). Underlying data can be found in S1 Data. AMPK, 5′ adenosine monophosphate-activated protein kinase; ANOVA, analysis of variance; dcr2, dicer-2; GAL4, galactose-responsive transcription factor; LHLK, Lateral Horn leucokinin; Lk, leucokinin; *luc*, *luciferase*; RNAi, RNA interference; UAS, upstream activation sequence.
(TIF)

S1 Data This file includes the raw data for each experiment within the main and supplemental figures for all genotypes and under fed and starved conditions. In addition, mean values and statistical analyses are included in the file for all conditions described.
(XLSX)

## Abbreviations

2DG: 2 deoxy-glucose
ABLK: abdominal leucokinin
AKH: adipokinetic hormone
ALK: anterior leucokinin
AMMC: antennal mechanosensory and motor center
AMPK: 5′ adenosine monophosphate-activated protein kinase
ANOVA: analysis of variance
Apt: *Apterous*
CNS: central nervous system
CRISPR/Cas9: Clustered Regularly Interspaced Short Palindromic Repeats/CRISPR-associated protein-9 nuclease
DAMS: *Drosophila* Activity Monitor System
dcr2: dicer-2
dFSB: dorsal fan-shaped body
DGRC: *Drosophila* Genomics Resource Center
Dilp2: *Drosophila* insulin-like peptide 2
elav: embryonic lethal abnormal vision
FoxO: forkhead box
GAL4: galactose-responsive transcription factor
GCaMP6m: GFP-calmodulin and M13 peptide sequence
GFP: green fluorescent protein
gRNA: guide RNA
HDR: homology-dependent repair
IHC: immunohistochemistry
impTNT: inactive variant of tetanus toxin
IPC: insulin-producing cell
IR: infrared light
LHLK: Lateral Horn leucokinin
Lk: leucokinin
*Lkr*: leucokinin receptor
mCD8::GFP: membrane-tethered green fluorescent protein
mTOR: mammalian target of rapamycin
nc82: neuropil marker
PBS: phosphate-buffered saline
PBST: phosphate-buffered saline, 0.5% Triton-X
RNAi: RNA interference
ROI: region of interest
SELK: subesophageal ganglion leucokinin
SIRT1: sirtuin-1
TNT: tetanus toxin
trsn: translin
tsh: teashirt
UAS: upstream activation sequence
UAS-Gerry: GCaMP6m-mCherry
ZT: Zeitgeber time
