# Supplementary figures and images for: A single pair of leucokinin neurons are modulated by feeding state and regulate sleep–metabolism interactions
Source: PLoS Biol. 2019 Feb 13;17(2):e2006409. doi: 10.1371/journal.pbio.2006409 (PMC6391015; doi:10.1371/journal.pbio.2006409)

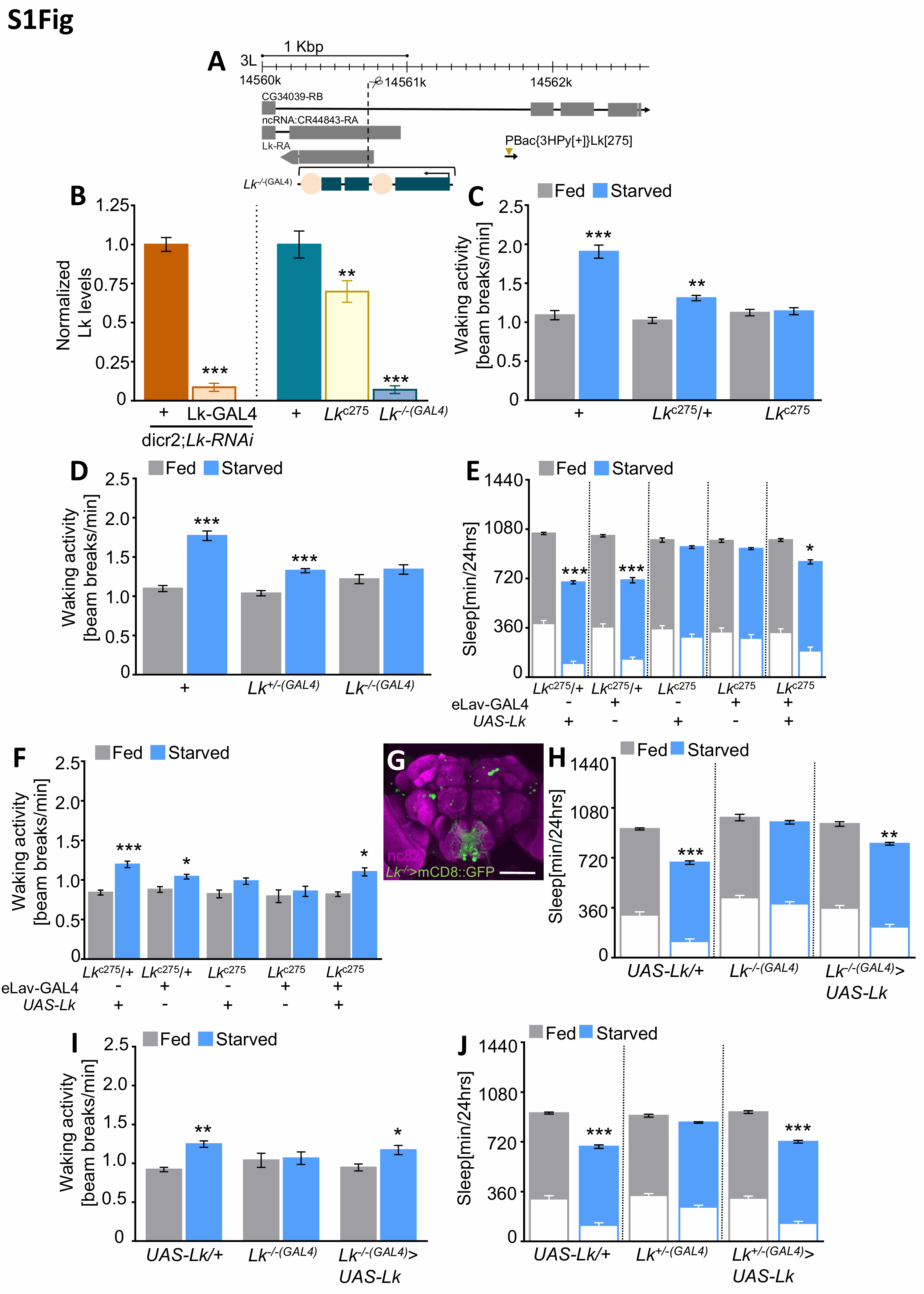

Supplement: S1 Fig — (A) The genomic organization of the Lk locus. Lkc275 consists of a piggyBac element inserted 929 base pairs 5′ to the transcription start site of the leucokinin gene (gold triangle). The dotted line corresponds to the cleavage site used for Lk−/−(GAL4) mutant generation by CRISPR/Cas9-mediated Lk genome engineering. Lk−/−(GAL4) contains a GAL4 element replacing base 1 to 7 downstream of the ATG site and a floxed 3xP3-RFP cassette (brackets, pale orange and turquoise). (B) Quantification of Lk peptide levels in LHLK neurons of dcr2,Lk-RNAi, Lkc275, and Lk−/−(GAL4) mutants. (C) Waking activity in Lkc275 mutants does not differ between the fed and starved states (n = 59, p > 0.99). Control flies (w1118, n = 64, p < 0.0001) and Lkc275/+ (n = 66, p = 0.0015) increase waking activity during starvation. Two-way ANOVA (F [2, 374] = 29.07). (D) Control flies (w1118, n = 77, p < 0.0001) and Lk+/−(GAL4) (n = 70, p = 0.0001) increase waking activity during starvation, while waking activity does not differ between the fed and starved states in Lk−/−(GAL4) (n = 47, p = 0.61). Two-way ANOVA (F [2, 382] = 17.47). (E) Pan-neuronal rescue of Lkc275 (elav-GAL4;Lkc275>UAS-Lk;Lkc275, n = 17, p = 0.04) restores starvation-induced sleep suppression compared to Lkc275 mutant controls UAS-Lk/+;Lkc275 (n = 24; p > 0.99) and elav-GAL4/+;Lkc275 (n ≥ 20, p = 0.99). Sleep duration on agar (starved) does not differ significantly between rescue and UAS-Lk/+;Lkc275/+ (n = 30, p = 0.08) or elav-GAL4/+;Lkc275/+ (n = 51, p = 0.11). Two-way ANOVA (F [4, 272] = 8.97). White bars in column graphs represent amount of sleep during the day (ZT 0–12), while colored bars represent night sleep (ZT 12–24). (F) Pan-neuronal rescue of Lkc275 (elav-GAL4;Lkc275>UAS-Lk;Lkc275) (n = 17, p = 0.02) restores starvation-induced increase in waking activity compared to Lkc275 mutant controls UAS-Lk/+;Lkc275 (n = 23, p = 0.37) and elav-GAL4/+;Lkc275 (n = 20, p > 0.99). No significant differences were seen during the starved [file pbio.2006409.s001.tif]

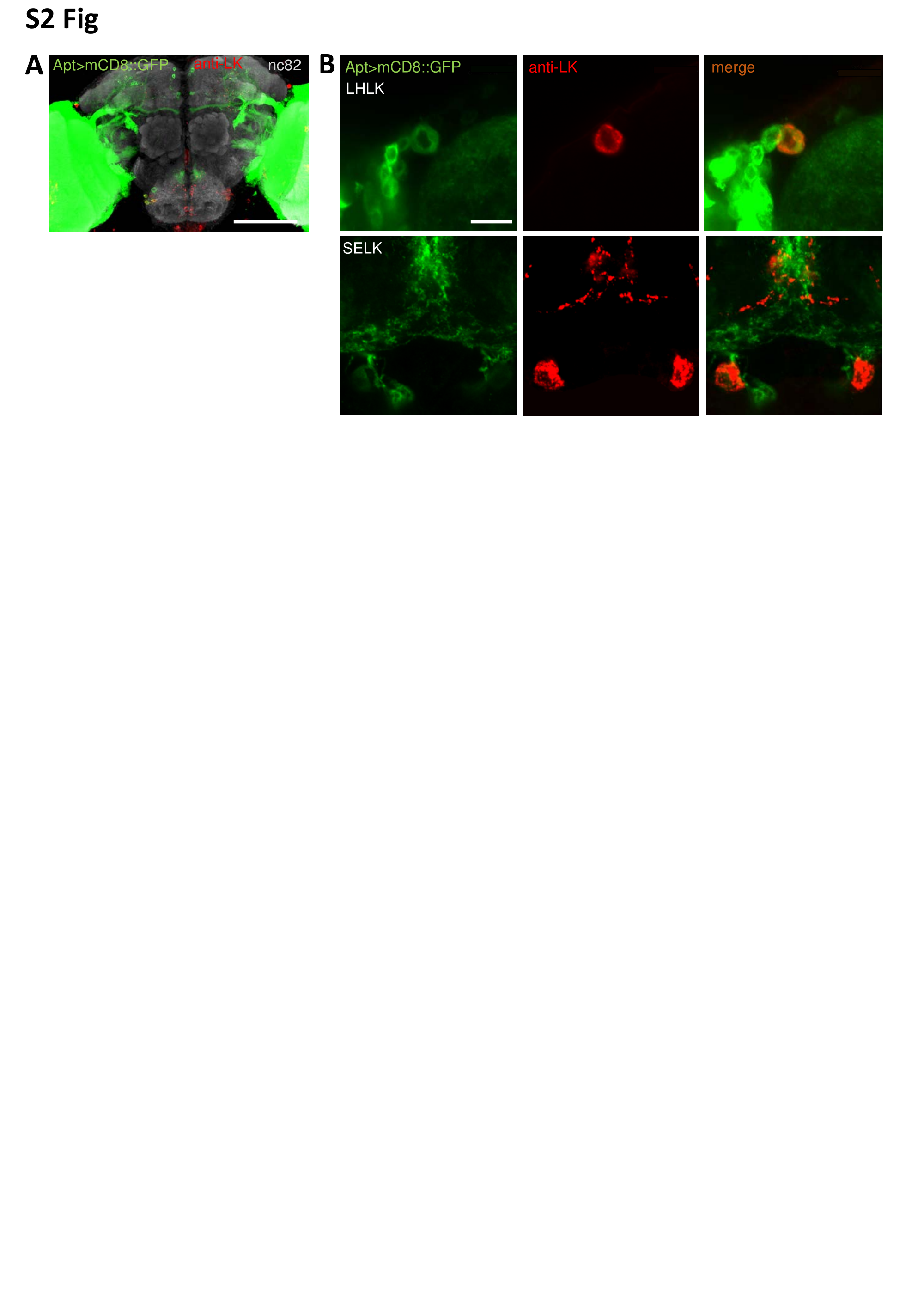

Supplement: S2 Fig — (A) Expression pattern of Apt-GAL4 driving mCD8::GFP (green) and endogenous expression of Lk neuropeptide (red). The brain was counterstained with nc82 (gray). Scale bar = 50 μm. (B) Immunostaining for anti-LK (red) in Apt-GAL4>mCD8::GFP (green) reveals LHLK localizes to neurons labeled by Apt-GAL4 (orange, top panel). SELK neurons (bottom panel) do not colocalize with SOG neurons labeled by Apt-GAL4. Depicted is a 14-μm section from the lateral horn region and a 6-μm section from the SOG region using a 60× oil immersion objective. Scale bar = 10 μm. Apt, apterous; CD8::GFP, LK-GAL4>CD8:GFP;tshGAL80; GAL4, galactose-responsive transcription factor; GFP, green fluorescent protein; LHLK, Lateral Horn leucokinin; Lk, leucokinin; mCD8::GFP, membrane-tethered GFP; nc82, neuropil marker; SELK, subesophageal ganglion leucokinin; SOG, subesophageal ganglion; tsh, teashirt. (TIFF) [file pbio.2006409.s002.tiff]

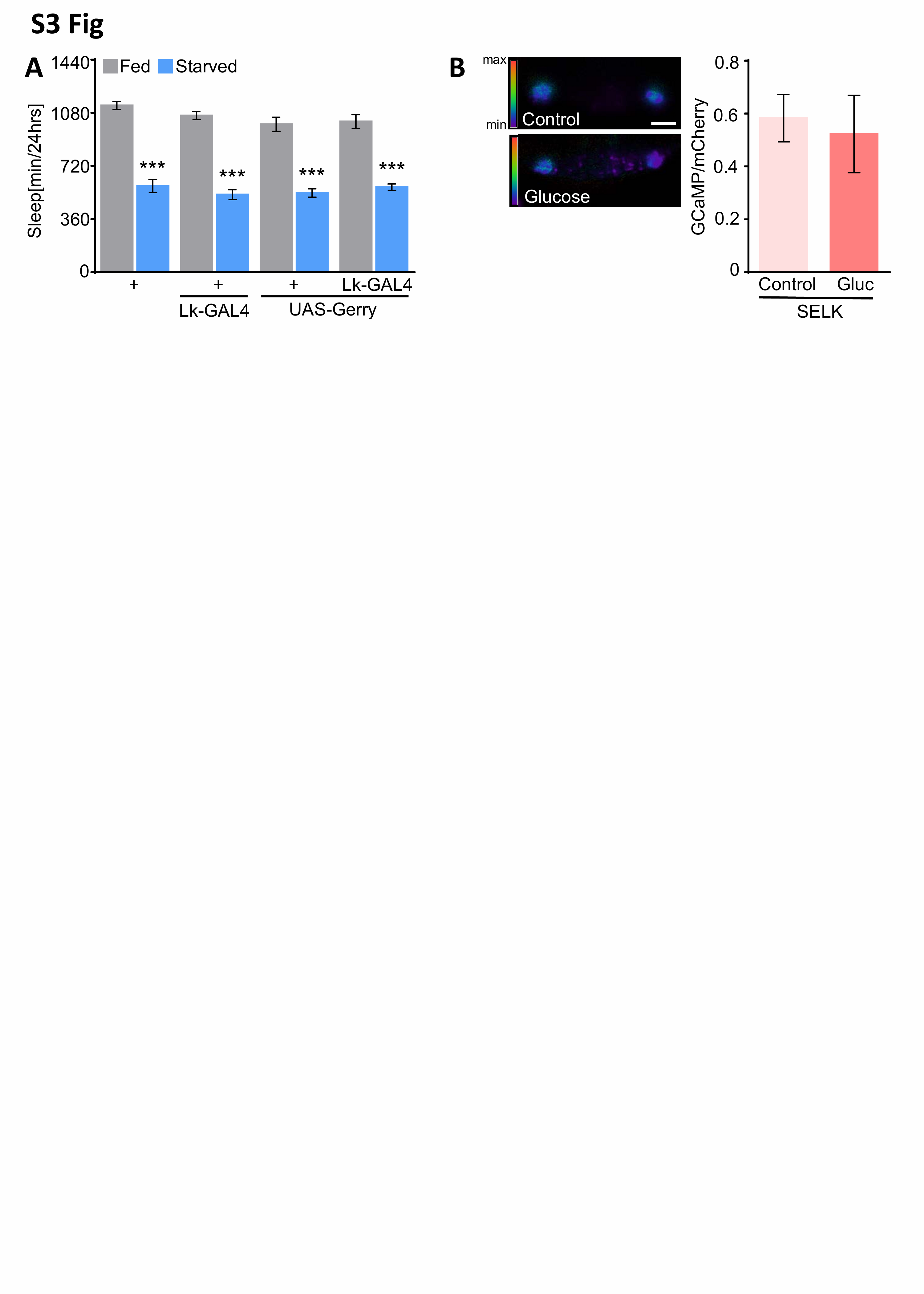

Supplement: S3 Fig — (A) Flies expressing UAS-Gerry in Lk-GAL4 sleep significantly more in food tubes (gray) than on agar (blue, n = 24, p < 0.0001) similar to control flies, UAS-Gerry/+ (n = 32, p < 0.0001), Lk-GAL4/+ (n = 31, p < 0.0001), or w1118 flies (n = 32, p < 0.0001). No significant differences were detected in the fed state between Lk-GAL4>UAS-Gerry and w1118 control (p = 0.45), UAS-Gerry alone (p > 0.99), or Lk-GAL4 alone (p = 0.99). Two-way ANOVA, (F [3, 230] = 0.97). All columns represent the mean ± SEM; ***p < 0.001. (B) No significant differences in GCaMP/mCherry were detected in SELK neurons in controls bathed with artificial hemolymph solution alone or 200 mM of glucose (n ≥ 4, p = 0.77, t = 0.3). Unpaired t test. Scale bar = 10 μm. Fluorescence intensity scale represents the ratio range of GCaMP6m/mCherry ranging from 5 (max) to 0 (min). Underlying data can be found in S1 Data. ANOVA, analysis of variance; GAL4, galactose-responsive transcription factor; GCaMP6m, GFP-calmodulin and M13 peptide sequence; LHLK, Lateral Horn leucokinin; Lk, leucokinin; max, maximum; min, minimum; SELK, subesophageal ganglion leucokinin; UAS, upstream activation sequence; UAS-Gerry, GCaMP6m-mCherry. (TIF) [file pbio.2006409.s003.tif]

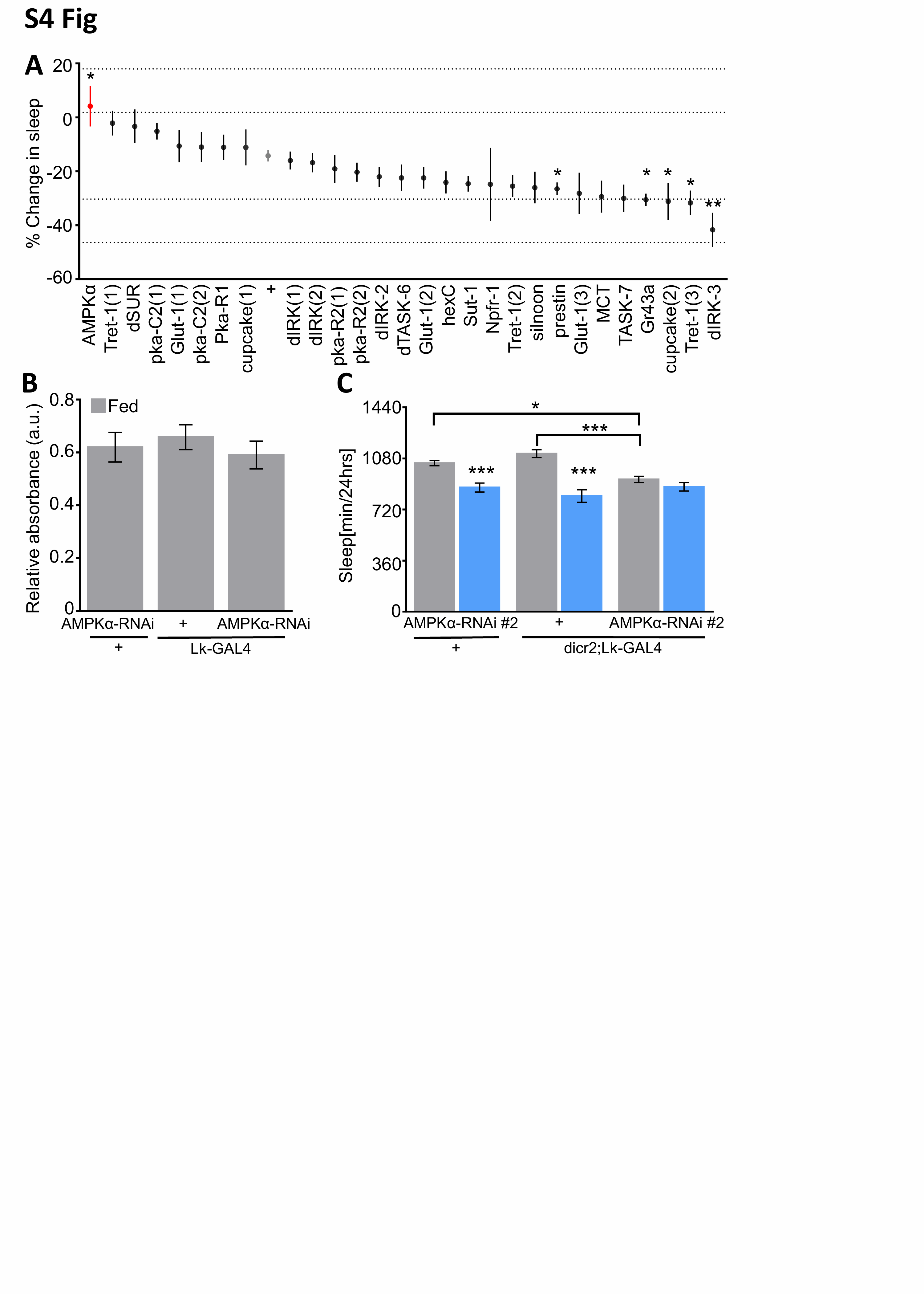

Supplement: S4 Fig — (A) The percentage change in sleep in an RNAi screen targeting nutrient sensors or signaling pathway molecules in Lk-GAL4 neurons. Greater sleep suppression was observed in controls Lk-GAL4 and the isogenic host strain for the Vienna Drosophila Resource Center RNAi library (Lk-GAL4/+, n = 56) compared to AMPKα-RNAi (n = 9, p = 0.04). One-way ANOVA with Dunnett, F (28, 358) = 4.47). Dashed lines indicate control mean ± 2 SD. (B) Knockdown of AMPKα in Lk neurons (Lk-GAL4> AMPKα-RNAi, n = 9) does not affect food intake during the fed state compared to AMPKα-RNAi/+ (n = 13, p = 0.65) and Lk-GAL4/UAS-dcr2,luc-RNAi (n = 12, p = 0.92). One-way ANOVA F (2, 31) = 0.40. (C) Expression of a second AMPKα-RNAi line in Lk neurons (Lk-Gal4>UAS-dcr2,AMPKα-RNAi) abolishes starvation-induced sleep suppression (n = 45, p = 0.76), while control flies dcr2,Lk-GAL4/+ (n = 32, p < 0.0001) and AMPKα-RNAi/+ (n = 44, p = 0.002) suppress sleep. In fed flies, sleep is significantly reduced in Lk-GAL4>UAS-dcr2, AMPKα-RNAi compared to Lk-GAL4/UAS-dcr2,luc-RNAi (p = 0.0004) and AMPKα-RNAi/+ (p = 0.04) controls. Two-way ANOVA (F [2, 236] = 8.89). Underlying data can be found in S1 Data. AMPK, 5′ adenosine monophosphate-activated protein kinase; ANOVA, analysis of variance; dcr2, dicer-2; GAL4, galactose-responsive transcription factor; LHLK, Lateral Horn leucokinin; Lk, leucokinin; luc, luciferase; RNAi, RNA interference; UAS, upstream activation sequence. (TIF) [file pbio.2006409.s004.tif]
